# Anaerobic degradation of 1-methylnaphthalene by a member of the *Thermoanaerobacteraceae* contained in an iron-reducing enrichment culture

**DOI:** 10.1007/s10532-017-9811-z

**Published:** 2017-11-24

**Authors:** Sviatlana Marozava, Housna Mouttaki, Hubert Müller, Nidal Abu Laban, Alexander J. Probst, Rainer U. Meckenstock

**Affiliations:** 1 Helmholtz Zentrum München, Institute of Groundwater Ecology, Ingolstädter Landstraße 1, 85764 Neuherberg, Germany; 2 Present Address: Intrapore UG, Katernberger Str. 107, 45327 Essen, Germany; 3 Biofilm Center, University of Duisburg-Essen, Universitätsstr. 5, 41451 Essen, Germany; 4 Group for Aquatic Microbial Ecology, Biofilm Center, University of Duisburg-Essen, Universitätsstr. 5, 41451 Essen, Germany

**Keywords:** Polycyclic aromatic hydrocarbons, 1-methylnaphthalene, *Thermoanaerobacteraceae*, *Desulfobulbaceae*, Iron reduction, Synthrophic degradation

## Abstract

An anaerobic culture (1MN) was enriched with 1-methylnaphthalene as sole source of carbon and electrons and Fe(OH)_3_ as electron acceptor. 1-Naphthoic acid was produced as a metabolite during growth with 1-methylnaphthalene while 2-naphthoic acid was detected with naphthalene and 2-methylnaphthalene. This indicates that the degradation pathway of 1-methylnaphthalene might differ from naphthalene and 2-methylnaphthalene degradation in sulfate reducers. Terminal restriction fragment length polymorphism and pyrosequencing revealed that the culture is mainly composed of two bacteria related to uncultured Gram-positive *Thermoanaerobacteraceae* and uncultured gram-negative *Desulfobulbaceae*. Stable isotope probing showed that a ^13^C-carbon label from ^13^C_10_-naphthalene as growth substrate was mostly incorporated by the *Thermoanaerobacteraceae*. The presence of putative genes involved in naphthalene degradation in the genome of this organism was confirmed via assembly-based metagenomics and supports that it is the naphthalene-degrading bacterium in the culture. *Thermoanaerobacteraceae* have previously been detected in oil sludge under thermophilic conditions, but have not been shown to degrade hydrocarbons so far. The second member of the community belongs to the *Desulfobulbaceae* and has high sequence similarity to uncultured bacteria from contaminated sites including recently proposed groundwater cable bacteria. We suggest that the gram-positive *Thermoanaerobacteraceae* degrade polycyclic aromatic hydrocarbons while the *Desulfobacterales* are mainly responsible for Fe(III) reduction.

**Electronic supplementary material:**

The online version of this article (10.1007/s10532-017-9811-z) contains supplementary material, which is available to authorized users.

## Introduction

**Fig. 1:**
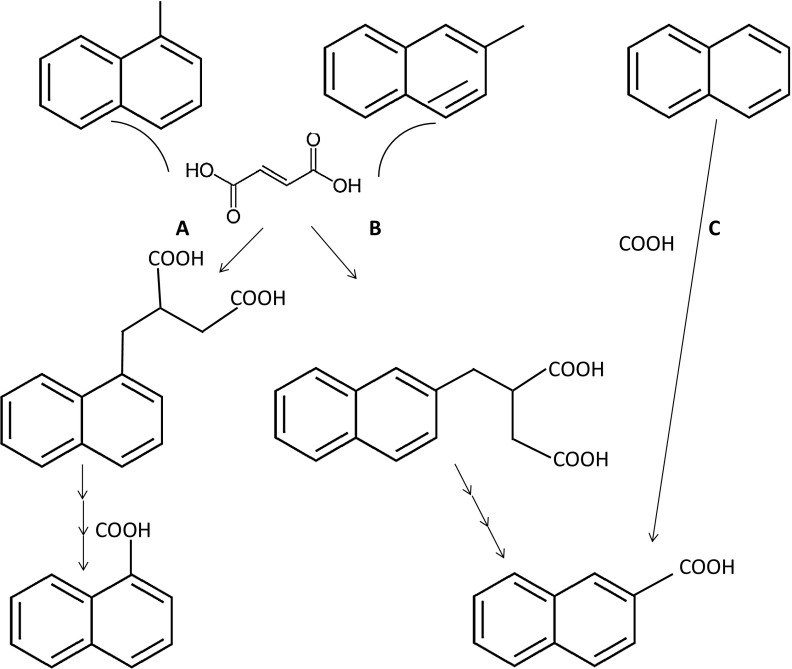
Activation mechanisms proposed for biodegradation of 1-methylnaphthalene (**a**) and 2-methylnaphthalene (**b**) via fumarate addition with subsequent formation of 1-naphthoic acid (**a**) and 2-naphthoic acid (**b**); and biodegradation of napthathalene (**c**) via carboxylation with formation of 2-naphthoic acid

Polycyclic aromatic hydrocarbons (PAHs) are frequent contaminants in groundwater and marine sediments due to accidents during crude oil production, transportation or storage of mineral oil products. Among those, naphthalene and 2-methylnaphthalene are in the top 30 of frequently occurring emerging pollutants according to the European Environment Agency groundwater organic micropollutant database (Stuart et al. 2012). Although indigenous microbial communities are able to degrade these compounds, their low water solubility, the chemical stability of the aromatic ring, and adsorption to sediments make PAHs poorly bioavailable. Due to low solubility of oxygen in water, oxygen is quickly removed by aerobic bacteria in sediments and groundwater upon contamination with hydrocarbons. Therefore, anaerobic utilization of PAHs is the prevailing process in contaminated aquifers (Meckenstock et al. 2015; Folwell et al. 2016). Anaerobic biodegradation of PAHs can be coupled to sulfate and iron reduction, and methanogenesis whereas reports on denitrifying cultures were not reproducible, so far (Meckenstock et al. 2016). The best investigated PAH-degrading cultures to date contain sulfate reducers (Galushko et al. 1999; Meckenstock et al. 2000; Musat et al. 2009) belonging to *Desulfobacteraceae* within the *Deltaproteobacteria* (Meckenstock and Mouttaki 2011). Incorporation of ^13^C-bicarbonate by a marine, sulfate-reducing enrichment indicated that naphthalene might be carboxylated to 2-naphthoic acid (Zhang et al. 2012a). Recently, it has been proven in biochemical studies with the highly enriched sulfate-reducing enrichment culture N47 originating from groundwater that naphthalene is indeed activated via carboxylation to 2-naphthoic acid (Mouttaki et al. 2012). Degradation of 2-methylnaphthalene is initiated via fumarate addition with formation of naphthyl-2-methyl-succinic acid (Fig. 1) (Annweiler et al. 2000). Then, naphthyl-2-methyl-succinic acid is converted to 2-naphthoic acid via several β-oxidation steps (Safinowski and Meckenstock 2004). 2-Naphthoic acid can be regarded as a central metabolite in the anaerobic degradation of naphthalene and 2-methylnaphthalene.

Ferric iron is a wide-spread electron acceptor in aquifers. Several pure cultures capable of biodegradation of monoaromatic hydrocarbons that have been isolated belong to *Geobacteraceae* (Lovley et al. 1993; Zhang and Young 1997; Coates et al. 2001; Nevin and Lovley 2002), *Rodocyclaceae* (Weelink et al. 2009), and *Peptococcaceae* (Kunapuli et al. 2010). Only recently, an iron-reducing, naphthalene-degrading enrichment culture has been described. It is dominated by members of the *Peptococcaceae* which can grow not only with naphthalene but also with 1-, and 2-methylnaphthalene as sole electron and carbon source (Kleemann and Meckenstock 2011).

Although both 1- and 2-methylnaphthalenes are readily degraded by aerobic cultures (Mahajan et al. 1994; Mueller‐Spitz and Crawford 2014), anaerobic degradation of 1-methylnaphthalene has been reported so far only for an anaerobic sediment enrichment (Genthner et al. 1997) or sludge (Christensen et al. 2004) under methanogenic conditions and for an iron-reducing enrichment N49 (Kleemann and Meckenstock 2011). In other studies, where 1-methylnaphthalene has been tested, growth was observed only on the isomer 2-methylnaphthalene (Meckenstock et al. 2000; Galushko et al. 2003; Musat et al. 2009). Therefore, 1-methylnaphthalene has been considered to be less susceptible to biodegradation and the degradation pathway of 1-methylnaphthalene remains unclear.

Here, we report on an iron-reducing enrichment which was cultivated with 1-methylnaphthalene as sole carbon and electron source. We performed stable isotope probing and assembly-based metagenome analysis of the 1MN culture grown with ^13^C_10_-naphthalene. The goal was to identify the PAH-degrading microorganisms in the culture and to obtain insight in the degradation process of 1-methylnaphthalene.

## Materials and methods

### Growth of enrichment culture

The 1-methylnapthalene-degrading culture 1MN was enriched from contaminated soil at a former coal gasification site in Gliwice, Poland with 1-methylnaphthalene as sole carbon source. 1-methylnaphthalene was provided as absorbed to a resin Amberlite XAD-7 (Morasch et al. 2001) and 50 mM Fe(OH)_3_ was added as electron acceptor. Fe(OH)_3_ was synthesized via neutralizing a 0.4 M solution of FeCl_3_ to a pH of 7 with NaOH (Lovley and Phillips 1988). The enrichment culture was cultivated in fresh water medium (Widdel and Bak 1991) (pH 7.2) and was reduced with 0.7 mM Na_2_S. 0.24 mM of the humic acid analogue 9,10-anthraquinone-2,6-disulfonic acid disodium salt was added in order to facilitate Fe(OH)_3_ reduction. Before carrying out the main experiments, the sediment-free cultures were transferred with 10% inoculum every 3 months over 6 years and cultivated in 100 ml serum bottles containing 90 ml of medium. In such a way, any carbon substrates associated with the source of isolation were eliminated from the culture medium.

In order to obtain a pure culture, serial dilutions to extinction were performed: inoculum was serially diluted in culture bottles up to 10^12^ dilution where no cells were expected to be. However, no pure cultures were obtained after.

For metabolite analyses, cultures were cultivated in 1 l bottles containing 900 ml medium. 1-Methylnaphthalene, 2-methylnaphthalene, or naphthalene, were dissolved as 50 mM solution in 2,2,4,4,6,8,8-heptamethylnonane (HMN). 1.4 ml from a 50 mM stock solution was added per 100 ml of culture medium.

For electron balance experiments, 10 µl (approximately, 11 mg or 0.08 mM) of 1-methylnaphthalene was added with a glass syringe directly into the culture bottles containing 900 ml autoclaved medium. Solubility of 1-methylnaphthalene at 25 °C is 25 mg l^−1^ (Linstrom and Mallard 2001) allowing for total dissolution of the 1-methylnaphthalene added. Bottles were shaken for three days at room temperature to fully dissolve the 1-methylnaphthalene prior to inoculation and no visible droplets of 1-methylnaphthalene remained in the liquid medium.

To test for sulfur cycling involved in iron reduction, culture 1MN was grown in media containing 30 mM of Fe(OH)_3_ reduced with 0.7 mM Na_2_S with 30 mM elemental sulfur in the absence of 1-methylnaphthalene and HMN. After 3 consecutive transfers, the culture was inoculated into freshwater medium with 30 mM Fe(OH)_3_ as electron acceptor, 30 mM elemental sulfur as electron donor, and 0.7 mM Na_2_S as a reducing agent in the absence of any organic carbon source. Sulfate, sulfide, and Fe(II) concentrations were monitored weekly and compared to abiotic controls.

**Fig. 2:**
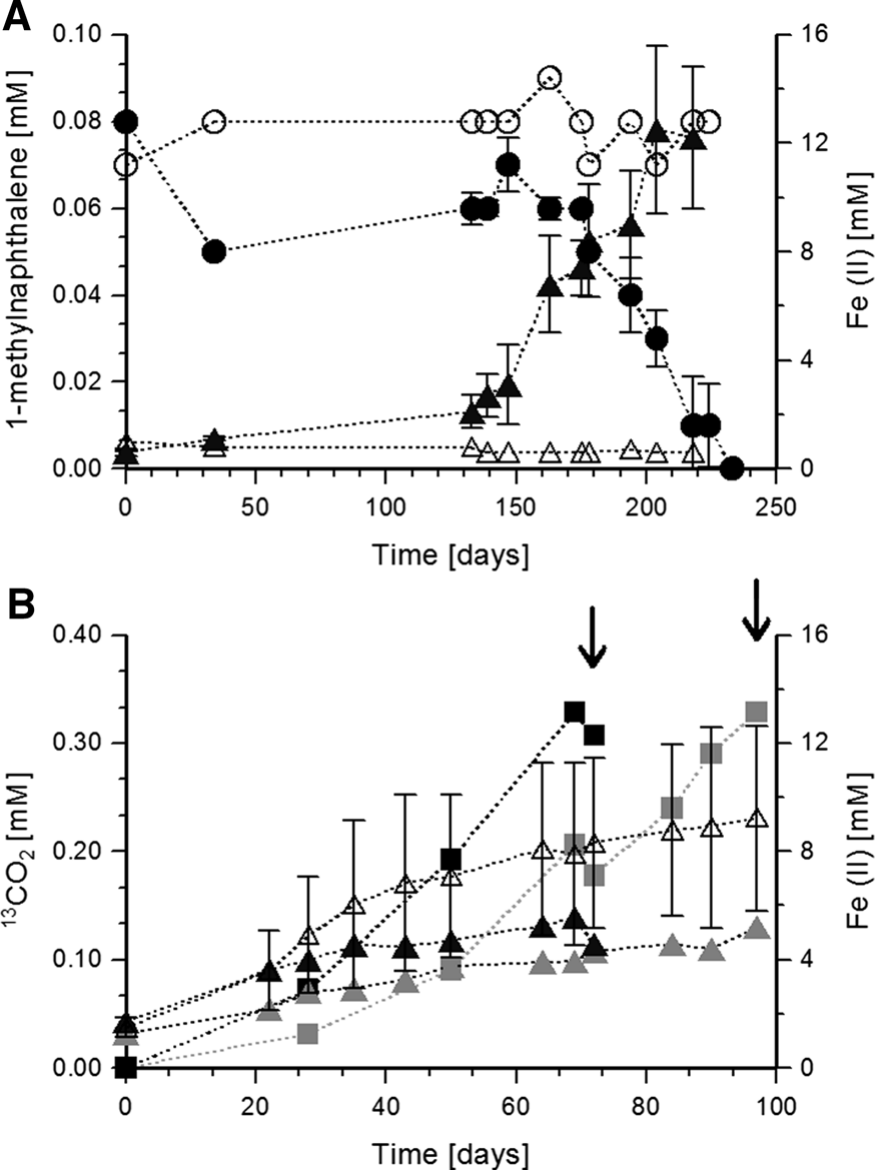
Degradation of polycyclic aromatic hydrocarbons by enrichment culture 1MN. **a** Growth of enrichment 1MN with 1-methylnaphthalene as carbon source and ferrihydrate as electron acceptor. Error bars represent standard deviation of two biological replicates. Filled circles, 1-methylnaphthalene concentrations in inoculated cultures; filled triangles, Fe(II) concentrations in inoculated cultures; open circles, 1-methylnaphthalene concentrations in abiotic control; open triangles, Fe(II) concentrations in abiotic control. **b** Growth with ^13^C_10_-naphthalene as carbon source and ferrihydrate as electron acceptor. Arrows indicate the days of sampling for stable isotope probing-analysis after 72 days of incubation with ^13^C_10_-naphthalene in early labeling experiment and 97 days of incubation with ^13^C_10_-naphthalene in late labelling experiment. Error bars represent standard deviations of two replicates. Filled triangles, Fe(II) concentrations in cultures with ^13^C_10_-naphthalene: black triangles—early harvested culture, grey triangles—late harvested culture; open triangles, Fe(II) concentrations in control cultures with ^12^C_10_-naphthalene; squares, ^13^CO_2_ concentration: black squares—^13^CO_2_ in early harvested culture; grey squares—^13^CO_2_ in late harvested culture

Stable isotope probing experiments were performed with fully labelled ^13^C_10_-naphthalene or ^12^C_10_-naphthalene (99% atoms Sigma-Aldrich) dissolved in HMN with final concentration of 0.05 and 0.07 mM, respectively. 10% of inoculum from cultures pre-grown with ^12^C_10_-naphthalene was added into 200 ml serum bottles filled with 170 ml of medium. Each condition was performed in duplicates. Growth was monitored via measuring Fe(II) production. For DNA extraction, each bottle with ^13^C_10_-naphthalene was harvested completely when approximately 0.3 mM CO_2_ equivalent to consumption of 0.03 mM naphthalene was produced in the 1st and 2nd bottle after 72 and 97 days, respectively (Fig. 2). Each control bottle with ^12^C_10_-naphthalene was harvested twice at the same sampling times of 72 and 97 days.

### Chemical analysis

Fe(II) was determined by the ferrozine assay according to (Braunschweig et al. 2012) using a Cary 50 Bio UV–Vis photometer (Varian, Darmstadt, Germany) at a wavelength of 508 nm. Sulfate was measured by ion chromatography on a Dionex Aquion system (Thermo Fisher Scientific, Dreieich, Germany). Sulfide was determined by the methylene-blue method which was downscaled to 96-well-plate format (Cline 1969; Müller et al. 2016).

1-Methylnaphthalene, 1-, and 2-naphthoic acids concentration was measured by HPLC (Shimadzu, Japan) on a PFP Kinetex column (75 × 4.6 mm) (Phenomenex Inc., USA). For 1-methylnaphthalene detection, a 40–60% gradient of acetonitrile in deionized water was applied at a flow rate of 1 ml min^−1^ (UV detection at 223 nm). 1-, and 2-naphthoic acids were separated by isocratic elution with 1% acetic acid in deionized water (solvent A) and acetonitrile (solvent B) (35:65, v:v) at a flow rate of 0.4 ml min^−1^ (UV detection at 224 nm). Column temperature was set to 30 °C.

### Metabolite analysis

For metabolite analysis, 300 ml aliquots were taken from cultures, adjusted to pH 12 with 1 N NaOH and stirred for 30 min to hydrolyze possible thioester bonds. Samples were acidified to pH < 2 with 12 N HCl, extracted three times with ethylacetate (1:1 ratio of sample to ethylacetate), and filtered through anhydrous sodium sulfate to remove traces of water. The collected ethylacetate phase was concentrated in a vacuum rotator at 65 °C to a volume of 2 ml and further evaporated under a stream of nitrogen gas to 1 ml. Samples were derivatized with 250 µl of N,O-bis-(trimethylsilyl)trifluoroacetamide (BSTFA) at 65 °C for 30 min.

The detection of the metabolites was performed with GC–MS (Agilent Technologies, GC System 7890A) equipped with a DB-5 column (0.25 μm film thickness, 0.25 mm i.d., 30 ml length, Agilent Technologies, USA). Sample injection was splitless (1 μl), and the flow rate of the helium carrier gas was 1.48 ml min^−1^. The oven temperature was 80 °C for 5 min, then ramped at a rate of 20 °C min^−1^ to 230 °C, and held for 10 min. The injector temperature was 270 °C.

For metabolite analysis with LC/MS/MS, 1 ml of culture was centrifuged at 25,000×*g* for 10 min. 150 µl aliquots of the resulting supernatants were transferred into small GC glass vials with a 200 µl insert. Metabolite analysis was carried out by LC/MS/MS on an Agilent 1200 series HPLC system coupled to an Applied Biosystems Q-Trap mass spectrometer equipped with a TurboSpray ionization source. Samples of 50 µl were injected to a LiChroCART^®^ 125-2 Purospher^®^ STAR RP-18e (5 μm) HPLC cartridge (Merck, Darmstadt, Germany). The column oven was set to 35 °C. A gradient of 25–90% acetonitrile in 0.1% acetic acid was run at a flow rate of 0.3 ml hour^−1^ over 30 min. The sample was infused into the mass spectrometer via multiple reaction monitoring in negative mode and an entrance potential of − 7 V. The declustering potential was set to − 40 V and the collision energy was adjusted to − 13 V.

### GC-C-IRMS analyses

The total amount of ^13^CO_2_ produced during degradation of ^13^C_10_-naphthalene was determined by measuring the ^13^CO_2_/^12^CO_2_ in the headspace of cultures cultivated with 0.05 mM of ^13^C_10_-naphthalene dissolved in HMN. The headspace sample (500 µl) was added to a 10 ml serum vial filled with helium, capped with blue butyl rubber stopper and stored at 4 °C until the analysis. The headspace samples were taken in triplicates. Gas samples (100 µl) were taken from the storage bottles and injected manually into a GC/C/IRMS system consisting of a TRACE GC Ultra gas chromatograph with split/splitless injector (GC) (Thermo Fischer Scientific, Milan, Italy) coupled to a Finnigan MAT 253 Isotope ratio mass spectrometer (IRMS) via Finnigan Gas combustion III interface (Thermo Fischer Scientific, Bremen, Germany). The GC was equipped with a RT-Q Plot^TM^ column (30 m length, 0.32 mm internal diameter). Helium served as a carrier gas with a constant flow rate of 1.4 ml min^−1^. The initial oven temperature was set at 50 °C and ramped at a rate of 20 °C min^−1^ to 230 °C and held for 5 min. The ^13^CO_2_ was calculated according to (Kleemann and Meckenstock 2011).

### ATP measurement

ATP concentrations were used to evaluate live bacterial biomass in the enrichment culture according to the modified protocol of (Hammes et al. 2010). The measurements were performed with the BacTiter-Glo™ Microbial Cell Viability Assay kit (Promega, Germany). In order to remove iron from the samples, 1 ml samples were spun down shortly for 10 s at 25,000×*g*. In order to lyse the cells, 50 µl of BacTiter-Glo™ reagent were added to a 38 °C preheated supernatant. After 1 min of incubation in a thermomixer (Eppendorf, Germany) at 38 °C and 500 rpm, luminescence was measured on a luminometer (Glomax, Turner Biosystems, Sunnyvale, CA). The data were recorded in the form of relative light units (RLU) and transferred into ATP concentrations (µM) via a calibration curve with ATP standards (Roche, Mannheim, Germany).

### Molecular analysis

For DNA extraction, at least 10 ml of samples were centrifuged for 10 min at 25,000×*g* and washed with 1× phosphate-buffered saline (PBS). Genomic DNA was extracted with a FastDNA Spin Kit for Soil (MP Biomedicals, Illkirch, France) and stored at − 20 °C until further analysis.

Bacterial 16S rRNA gene-targeted terminal restriction fragment length polymorphism (T-RFLP) fingerprinting was performed using Ba27f–FAM/907r primers. FAM-labelled amplicons were generated as described in (Pilloni et al. 2011), digested with the restriction enzyme MspI (Promega, Mannheim, Germany) and separated by capillary electrophoresis on ABI 3730 48-capillary sequencer as described in (Lueders et al. 2006).

Cultures which were cultivated for 1 month and showed bacterial activity via AQDS reduction (visual observation of yellow color) with 1-methylnaphthalene dissolved in HMN were used for pyrosequencing analysis. Barcoded amplicons for multiplexing were prepared with the primers Ba27f (5′-aga gtt tga tcm tgg ctc ag-3′) and Ba519r (5′-tat tac cgc ggc kgc tg-3′) (Lane 1991) extended as amplicon fusion primers with respective primer A or B adapters, key sequence and multiplex identifiers (MID). Amplicon pyrosequencing on a 454 GS FLX Titanium system (Roche, Penzberg, Germany) and pyrotag data handling were performed according to (Pilloni et al. 2012). The generated data were assembled to contigs via the SEQMAN II software (DNAStar, Madison, WI), using forward- and reverse-reads, as described in (Pilloni et al. 2011). Created contigs were used for in silico T-RF prediction by TRiFLe (Junier et al. 2008). All assembled contigs from this study were deposited with GenBank under the accession numbers KY417998–KY418001. Classification of created contigs was performed with the RDP naïve Bayesian Classifier (Wang et al. 2007). Phylogenetic trees were created with the MEGA version 6 software using a maximum likelihood method (Hall 2013).

### Assembly-based metagenomics

For metagenome sequencing, we used a subsample of DNA extracted from culture 1MN grown with ^13^C_10_-naphthalene and harvested after 97 days of cultivation. The same sample was also used for the gradient centrifugation of the SIP experiments. Library preparation and paired-end Illumina HiSeq sequencing (read length 150 bp) were performed at GATC (Konstanz, Germany). Quality filtered reads [bbduck (http://jgi.doe.gov/data-and-tools/bbtools/) followed by SICKLE (Version 1.21, https://github.com/najoshi/sickle)] were assembled using metaSPADES version 3.10.1 using default settings (Nurk et al. 2017). Genes were predicted using prodigal in the meta mode (-p meta) (Hyatt et al. 2010). Scaffolds were classified against UniRef100 (Suzek et al. 2007) using diamond blastp (Buchfink et al. 2015) by retrieving the taxonomy of the best blast hit for each protein of the scaffolds (e-value 10E−5) followed by the calculation of the lowest taxonomic rank that covered at least 50% of the proteins present on the scaffold. Proteins were screened for naphthyl-2-methylsuccinate synthase (NmsA) and naphthalene carboxylase genes using diamond blastp (Buchfink et al. 2015) using previously published sequences retrieved from the non-redundant NCBI protein database as a template. Scaffolds carrying genes of interest were checked manually for scaffolding errors by mapping reads (Langmead and Salzberg 2012); default settings followed by visual inspection.

### Gradient centrifugation of extracted labeled and unlabeled DNA of SIP experiment

The DNA harvested from labelled and control experiments was quantified by Quant-iT PicoGreen dsDNA quantification kit (Invitrogen, Paisley, UK). At least 600 ng of DNA was loaded on a gradient buffer of CsCl (average density 1.71 g ml^−1^, Calbiochem, Merck, Darmstadt, Germany) in gradient buffer (0.1 M Tris–HCl at pH 8, 0.1 M KCl, 1 mM EDTA) and centrifuged (180,000×*g*, at 20 °C for > 36 h) as described in detail in (Lueders et al. 2004). The gradient centrifugation was performed in 5.1 ml polyallomer quick seal tubes in a VTI 65.2 vertical rotor using a Centrikon T-2190 centrifuge (Kontron Instruments, Milano, Italy). The steps after centrifugation were performed according to (Lueders 2010). Briefly, each gradient was divided into 13 equal fractions with ‘heavy’ DNA at the bottom and ‘light’ DNA at the top of the tubes using a Perfusor V syringe pump (Braun, Melsungen, Germany). Aliquots of 100 µl were used to determine the density of each gradient fraction using an AR200 digital refractometer (Reichert Inc., Depew, NY, USA). DNA was retrieved from each fraction with polyethylene glycol precipitation, washed in 70% ethanol and re-eluted in 30 µl elution buffer (Qiagen, Hilden, Germany). Bacterial 16S rRNA genes were quantified via qPCR from each precipitated fraction as described in (Kunapuli et al. 2007). Terminal restriction fragment length polymorphism (T-RFLP) fingerprinting was done for the six fractions with the most of DNA (97% of all recovered DNA).

## Results

### Degradation of 1-methylnaphthalene and other carbon sources

The iron-reducing culture 1MN was enriched from contaminated soil with 1-methylnaphthalene. After approximately 10 transfers, regular T-RFLP fingerprinting showed a stable microbial community consisting of three major tRFs at 149, 160, and 215 bp. The culture was routinely cultivated with 1-methylnaphthalene dissolved in 2,2,4,4,6,8,8-heptamethylnonane (HMN). Besides 1-methylnaphthalene degradation (Fig. 2a), the culture grew with the following substrates: 2-methylnaphthalene, naphthalene, 1- and 2-naphthoic acids (Table 1). The culture growth was monitored via analysis of Fe(II) production as well as visual inspection of the development of yellow color due to biological AQDS reduction. When nitrate or sulfate were used as electron acceptors instead of Fe(OH)_3_ with 1-methylnaphthalene as a carbon source, no bacterial growth was observed as there was no change in optical density over the course of cultivation. No AQDS reduction was observed when HMN only was provided without any of the above-mentioned carbon sources. No degradation of methylnaphthalenes in uninoculated bottles with Fe(OH)_3_ was detected. For example, Fig. 2a shows that in abiotic control there was no decrease in 1-methylnaphthalene concentration as well as no Fe(II) production over incubation time.

**Table 1:** List of the alternative substrates tested for growth of the 1-methylnaphthalene-degrading enrichment

Substrates tested	Concentration (mM)	Growth
1-Methylnapthalene	0.07	+
2-Methylnapthalene	0.07	+
Naphthalene	0.07	+
1-Naphthoic acid	0.1	+
2-Naphthoic acid	0.1	+
1-Naphthyl acetate	0.1	–
2-Napthyl acetate	0.1	–
1-Napththol	0.1	–
2-Naphthol	0.1	–
Anthracene	0.8	–
Phenanthrene	0.7	–
Benzofuran-1	0.7	–
Toluene	0.5	–
Benzoate	1	–
Puryvate	1	–
Hydroxybenzoate	0.5	–

To determine the electron balance, the culture was transferred to medium with 0.08 mM 1-methylnaphthalene dissolved in the aqueous phase. The degradation of 1-methylnaphthalene has occurred in two stages. In the first long stage, it took 138 days to degrade 0.021 mM of 1-methylnaphthalene and produce 3.5 and 1.6 mM of Fe(II). Such a long phase could be explained by adaptation of bacterial cells to relatively high concentration of 1-methylnaphthalene in the water as the pre-culture has been cultivated in the medium where 1-methylnaphhtalene has been dissolved in HMN phase. The second stage of degradation has been characterized by consumption of 0.06 mM of 1-methylnaphthalene within 91 days and production of 9.9 and 8.2 mM of Fe(II) (Fig. 2a). According to stoichiometric calculation (Eq. 1), 0.08 mM of 1-methylnaphthalene consumed would lead to the production of 4.5 mM of Fe(II). However, in this experiment 13.3 and 9.8 mM of Fe(II) were produced. Even though 1-methylnaphthalene degradation alone does not explain this high amount of Fe(II) production, these results indicate that biological degradation of 1-methylnaphthalene was detected. In biological replicates, 1-methylnaphthalene has been degraded completely while in abiotic control concentrations remained without change (Fig. 2a).

1 $${\text{C}}_{ 1 1} {\text{H}}_{ 10} + {\text{ 54 Fe}}\left( {\text{OH}} \right)_{ 3} \to 1 1 {\text{ HCO}}_{ 3}^{ - } + {\text{ 54 Fe}}^{ 2+ } + {\text{ 32 H}}_{ 2} {\text{O }} + {\text{ 97 OH}}^{ - }$$

This was further supported by cultivation with 0.05 mM of ^13^C_10_- naphthalene dissolved in HMN to prove the mineralization to CO_2_ (Fig. 2b). In two replicate bottles, 0.3–0.34 mM of ^13^CO_2_ were produced after 72–97 days of cultivation (Fig. 2b). The amount of ^13^CO_2_ produced equaled to 0.03–0.034 mM of naphthalene consumed demonstrating a total oxidation to CO_2_ and clear indication of bacterial degradation of ^13^C_10_-naphthalene. According to the stoichiometry of complete mineralization of naphthalene to CO_2_ (Eq. 2.), 0.03–0.034 mM of consumed naphthalene would require 1.44 mM of Fe(III) reduction. However, 2.8–3.9 mM of Fe(II) was detected suggesting an electron recovery of over 200%.

2 $${\text{C}}_{ 10} {\text{H}}_{ 8} + {\text{ 48 Fe}}\left( {\text{OH}} \right)_{ 3} \to 10{\text{ HCO}}_{ 3}^{ - } + {\text{ 48 Fe}}^{ 2+ } + {\text{ 28 H}}_{ 2} {\text{O }} + {\text{ 86 OH}}^{ - }$$

**Fig. 3:**
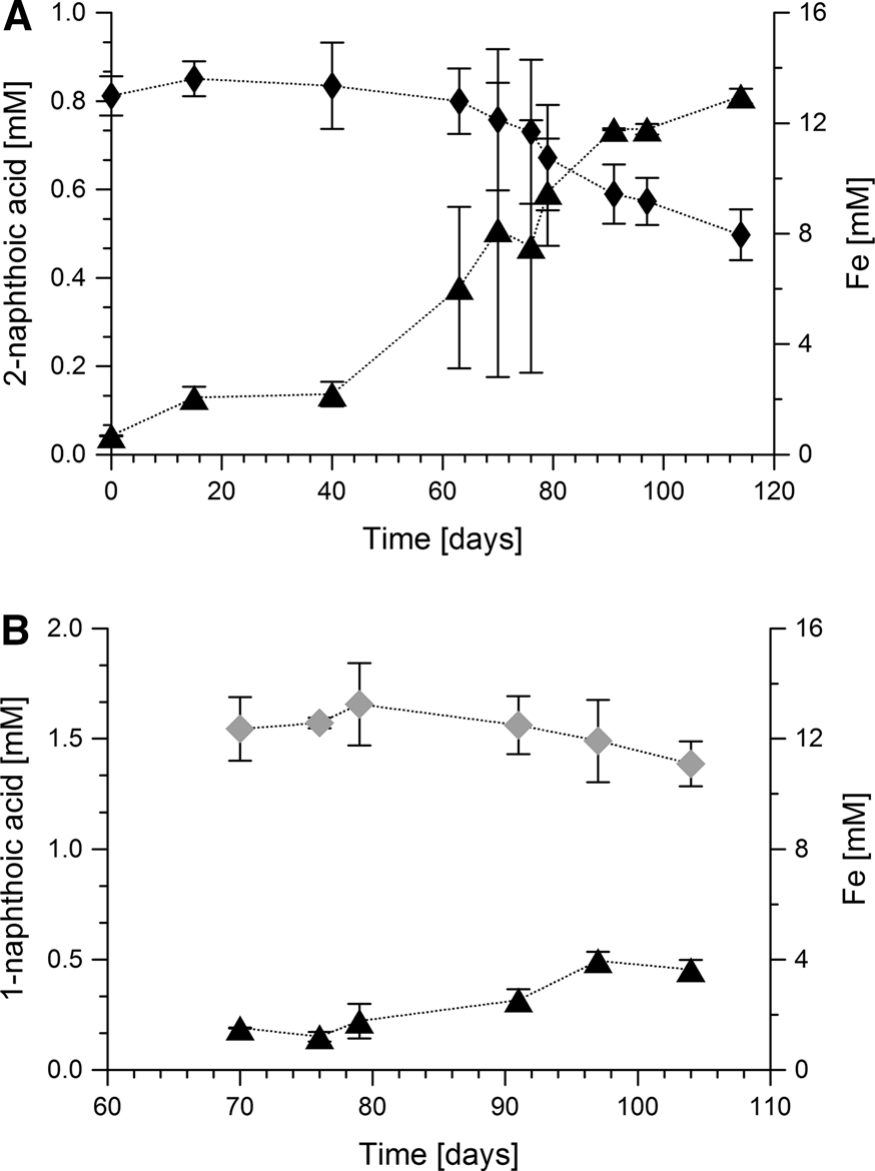
Growth of the 1-methylnaphthalene degrading enrichment 1MN with 2-naphthoic acid (**a**) and 1-naphthoic acid (**b**). Error bars indicate standard deviations of two biological replicates. Filled triangles, Fe(II); black diamonds, 2-naphthoic acid; grey diamonds, 1-naphthioc acid

The 1-methylnaphthalene-degrading enrichment was also cultivated with 1-, and 2-naphthoic acids (Fig. 3). Degradation of 315 ± 57 µM of 2-naphthoic acid within 97 days was coupled to production of 12.3 mM of Fe(II) (Fig. 3a). The 1-methylnaphthalene-degrading enrichment consumed 160 ± 43 µM of 1-naphthoic acid within 34 days and produced 3.0 ± 0.32 mM of Fe(II) (Fig. 3b). According to the stoichiometric equation Eq. (3), consumption of 315 µM of 2-naphthoic acid and 160 µM of 1-naphthoic acid should be coupled to production of 15 mM and 7.6 mM of Fe(II), respectively. However, 2.8 or 4.5 mM of Fe(II) were not recovered.

3 $${\text{C}}_{ 1 1} {\text{H}}_{ 8} {\text{O}}_{ 2} + {\text{ 48 Fe}}\left( {\text{OH}} \right)_{ 3} \to 1 1 {\text{HCO}}_{ 3}^{ - } + {\text{ 48 Fe}}^{ 2+ } + {\text{ 28 H}}_{ 2} {\text{O }} + {\text{ 85 OH}}^{ - }$$

The experiments with 1-methylnaphthalene or naphthalene as carbon sources showed up to 300% more Fe(II) produced than expected indicating the presence of further electron sources in the culture medium. We added 0.7 mM Na_2_S as a reducing agent which would account for the reduction of 5.6 mM Fe(III) if fully oxidized to SO_4_^2−^ (Eqs. 4, 5). HS^−^ reacts spontaneously with Fe(III) producing ferrous iron and elemental sulfur (Eq. 4) (Poulton 2003; Hellige et al. 2012). The elemental sulfur could then be disproportionated by bacteria to sulfate and sulfide (Eq. 4) which again can be abiotically oxidized by Fe(OH)_3_ reduction (Thamdrup et al. 1993; Finster et al. 1998) (Eq. 5).

4 $$3 {\text{ HS}}^{ - } + {\text{ 2 Fe}}\left( {\text{OH}} \right)_{ 3} + {\text{ 3 H}}^{ + } \to 2 {\text{ FeS }} + {\text{ S}}^{0} + {\text{ 6 H}}_{ 2} {\text{O}}$$

5 $$4 {\text{ S}}_{0} + {\text{ 4 H}}_{ 2} {\text{O}} \to {\text{SO}}_{ 4}^{ 2- } + {\text{ 3 HS}}^{ - } + {\text{ 5 H}}^{ + }$$

In order to prove that a potential sulfur cycle could be involved in iron reduction, we grew the enrichment without any organic electron donors and only with 0.7 mM Na_2_S and 30 mM Fe(OH)_3_ as electron donor and acceptor, respectively. After 27 days of incubation about 1.2 mM Fe(II) and 0.3 mM sulfate were produced (Fig. S1). Both values are much higher than expected from sulfur disproportionation alone indicating a complete oxidation of elemental sulfur to sulfate via an intermediate oxidation of sulfide to sulfur by iron reduction. Involvement of a sulfur cycle is also supported by the fact that enrichment culture 1MN was not able to grow in the same culture medium where Na_2_S was replaced by FeCl_2_.

**Fig. 4:**
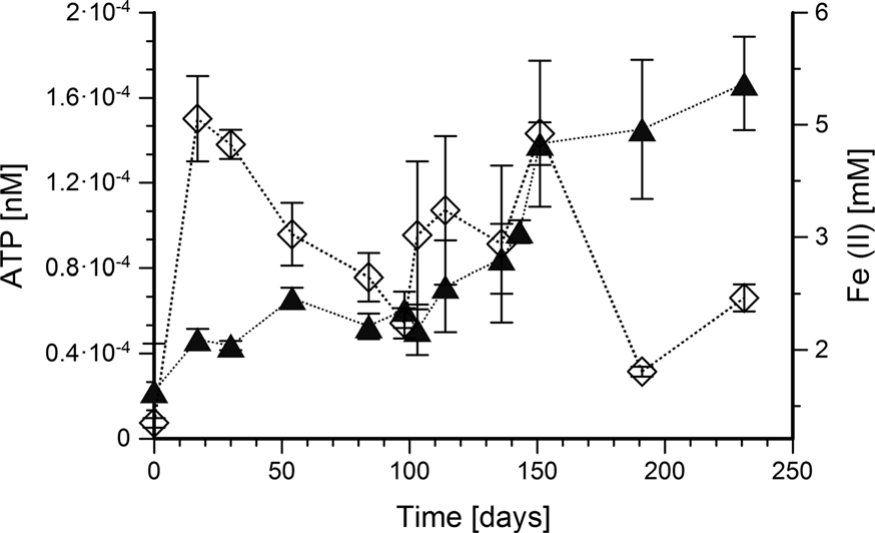
ATP production by the 1-methylnaphthalene-degrading enrichment 1MN. Error bars represent standard deviations of two biological replicates. Filled triangles, Fe(II) concentrations; open diamonds, ATP concentrations

In a separate experiment, the amount of ATP was analyzed as a measure of biomass and microbial growth with 1-methylnaphthalene. ATP was chosen to monitor cell numbers due to high amounts of solid Fe(OH)_3_ prohibiting microscopic cell counting. Presence of two ATP peaks indicated different time points with especially high activity (Fig. 4). The first high ATP peak indicates high metabolic activity at the start of growth. The second ATP peak corresponds to the steepest increase in Fe(II) after 100 days of cultivation. These two points of high activity might be related to initial sulfur disproportionation and subsequent active growth on 1-methylnaphthalene.

### Metabolites produced during growth with 1- and 2-methylnaphthalene and naphthalene

**Fig. 5:**
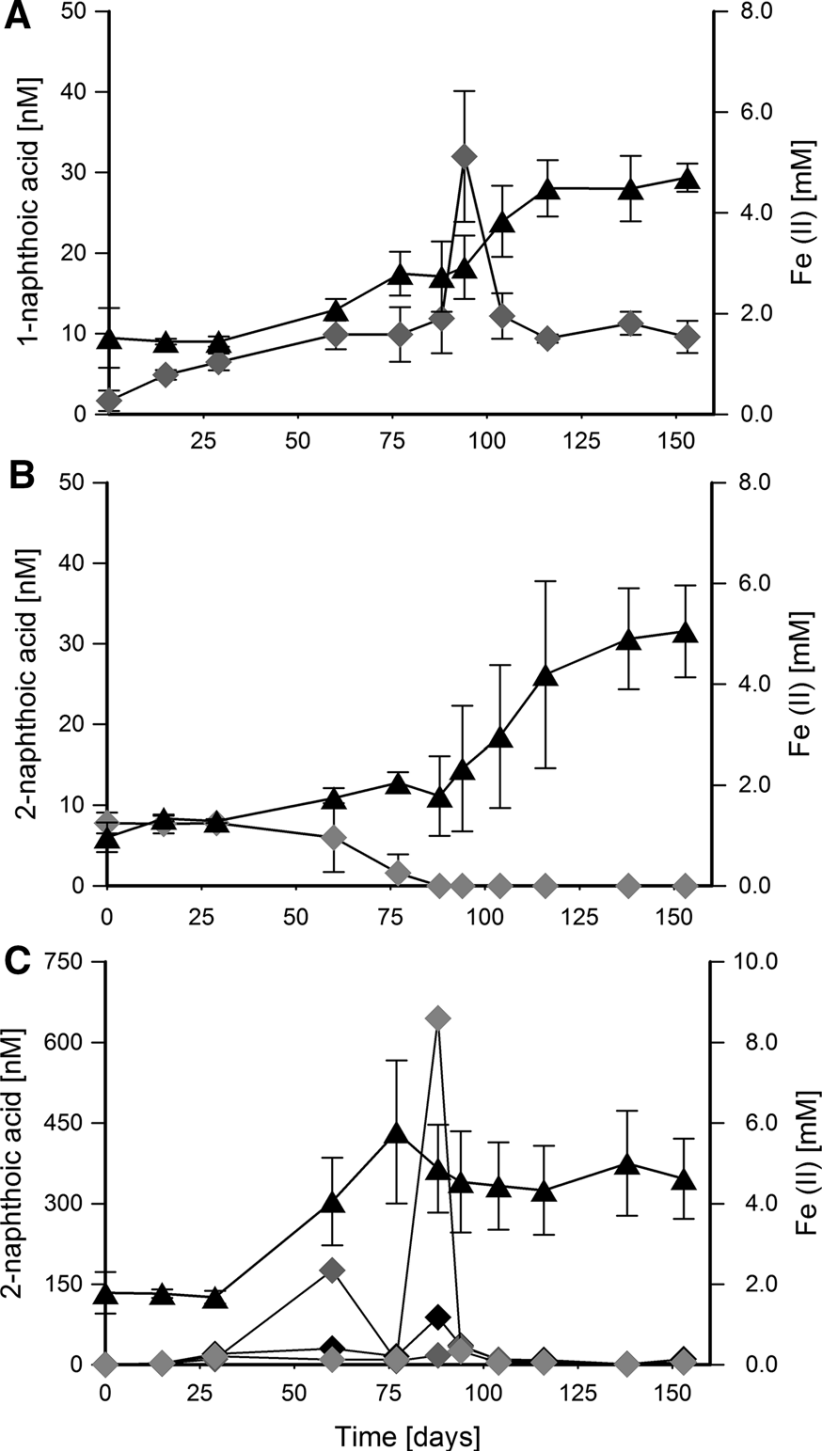
Production of Fe(II) and naphthoic acids by the 1-methylnaphthalene-degrading enrichment 1MN during growth with **a** 1-methylnaphthalene, **b** 2-methylnaphthalene, and **c** naphthalene. Error bars represent standard deviations of three biological replicates. In graph (**c**) measurements for naphthoic acids are not averaged because the production of the naphthoic acids strongly differed between the replicates. Filled triangles, Fe(II) concentrations; diamonds, naphthoic acid concentrations; different colors of diamonds in graph (**c**) represent individual biological replicates

When the culture was grown with 1-methylnaphthalene, 2-methylnaphthalene, or naphthalene, the following metabolites were detected in the culture medium: 1-naphthoic, 2-naphthoic, and 2-naphthoic acid, respectively (Fig. 5). The production of the metabolites was detected during growth, but was inconsistent over time (Fig. 5). The other putative acids from downstream degradation detected by GC–MS are mentioned in Supplementary material, Fig. S2.

### Analysis of the microbial community composition of enrichment culture 1MN

**Fig. 6:**
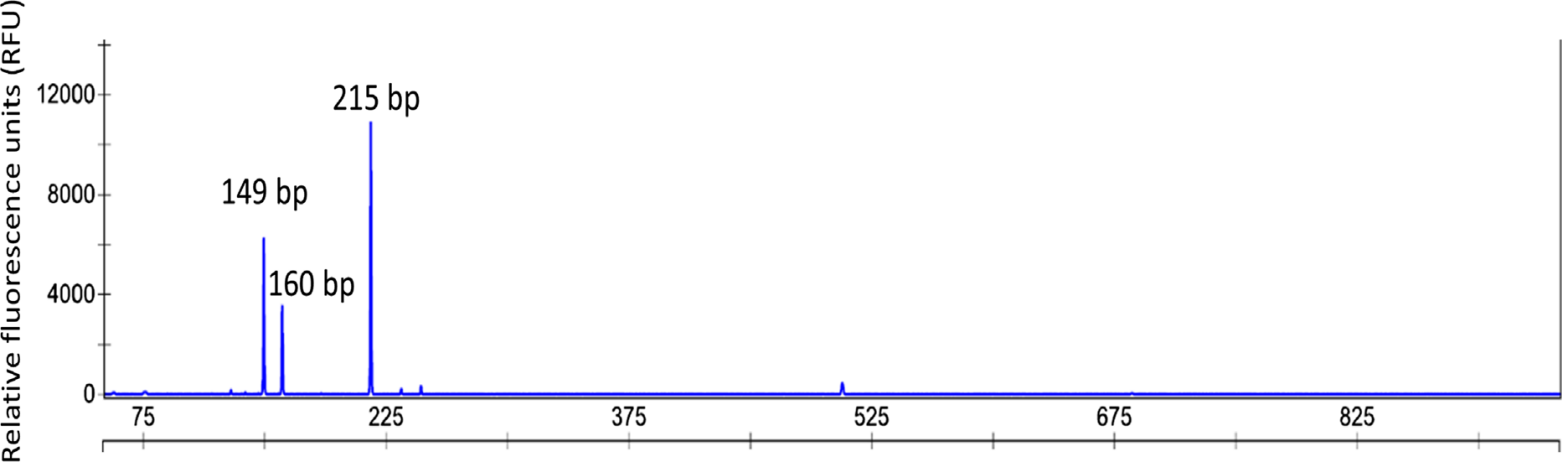
Electropherogram of a T-RFLP analysis of the 1-methylnaphthalene-degrading enrichment 1MN incubated with 1-methylnaphthalene as sole carbon and electron source. Numbers above the T-RF peaks give their length in base pairs

To identify the microorganisms involved in naphthalene degradation by culture 1MN, T-RFLP and sequence analyses of the 16S rRNA genes were performed. The electropherogram of 16S rRNA gene amplicons of the DNA extracted from cells grown with 1-methylnaphthalene showed three dominant TRF peaks of 149, 160, and 215 bp (Fig. 6). T-RFLP analyses of cultures incubated with 2-methylnaphthalene and naphthalene did not show significant changes in microbial composition indicating that the same microorganisms were responsible for the degradation (Supplementary data, Fig. S3). Only the abundance of the 160 bp peak decreased during later stages of degradation with naphthalene and 2-methylnaphthalene (Supplementary data, Fig. S3). However, when growing the culture in the absence of an organic carbon source only with the reducing agent 0.7 mM Na_2_S and 30 mM Fe(OH)_3_ as electron acceptor, the abundances changed drastically. TRFs 149 and 215 were not detectable anymore, whereas TRF 160 was highly enriched to more than 90% relative abundance in two replicate cultures (Fig. S1b).

**Fig. 7:**
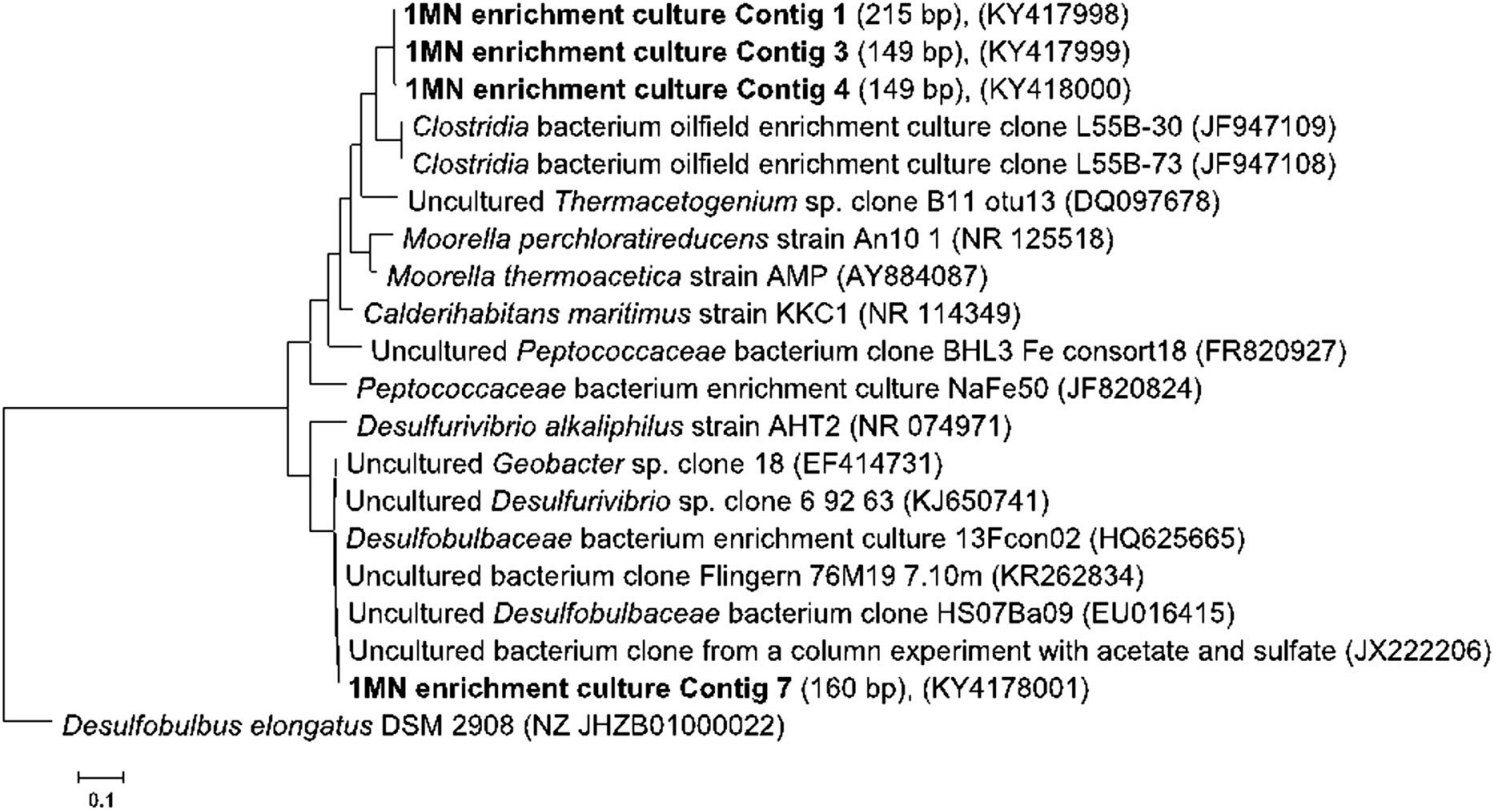
Phylogenetic tree created by maximum likelihood positioning of 16S rRNA gene sequences obtained from the 1-methylnaphthalene-degrading enrichment culture 1MN. The tree was rooted on *Desulfobulbus elongatus* DSM 2908 as outgroup. Sequences retrieved from the enrichment culture are depicted in bold. The bar indicates 10% estimated sequence divergence

To correlate the results of the T-RFLP analysis with the microbial phylogenetic affiliation, we performed pyrosequencing of 16S rRNA gene sequences with subsequent creation of contigs and in silico restriction. In general, 10 contigs were generated, four of them representing 90% of the community (Supplementary data, Table S1). The contigs corresponding to TRF peaks of 149 (contigs 3 and 4) and 215 bp (contig 1) were almost identical: contig 1 was similar to contigs 3 and 4 by 98.7% while contigs 3 and 4 were similar to each other by 98.4% (according to sequence identity matrix created in Bioedit). These three contigs represented together 62% of the community (Supplementary data, Table S1). According to the RDP Classifier, the contigs corresponding to TRF peaks 149 and 215 bp belong to *Clostridia* (95% classification reliability), *Thermoanaerobacterales* (84% classification reliability), *Thermoanaerobacteraceae* (84% classification reliability) and are distantly related (92% identical) to *Clostridial* gene clones from microorganisms enriched from oilfields under mesophilic and thermophilic conditions (Cheng et al. 2014) (Fig. 7). The sequence similarity of the 16S rRNA genes indicates that the contigs are from different strains of the same species. The other abundant contig (29% from all reads) corresponded to the 160 bp TRF peak and based on RDP classification belonged to the *Deltaproteobacteria, Desulfobacterales* (94% classification reliability), *Desulfobulbaceae* (65% classification reliability) and has 99% similarity to clones from an enrichment study where acetate was amended into sediment columns with sulfate as electron acceptor (Handley et al. 2013) (Fig. 7).

**Fig. 8:**
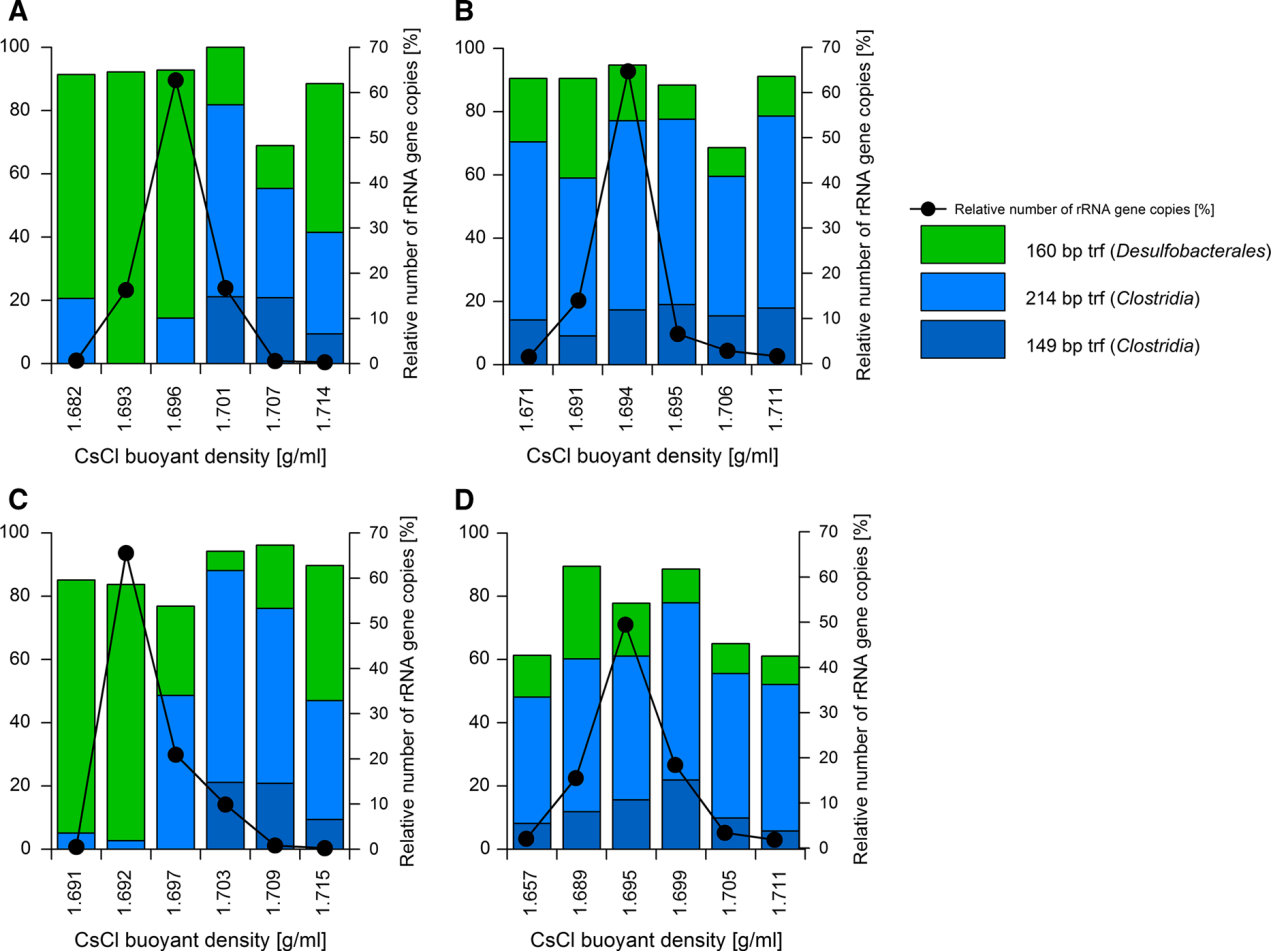
Relative T-RF abundances and relative number of rRNA gene copies distribution in comparative CsCl density-gradient centrifugations of DNA extracted from the 1-methylnaphthalene-degrading, iron-reducing enrichment 1MN incubated with either ^13^C_10_-naphthalene (**a**, **c**) or ^12^C_10_-naphthalene (**b**, **d**) at 72 h of cultivation (**a**, **b**) and 97 days of cultivation (**c**, **d**). Distribution of bacterial 16S rRNA genes within density gradient fractions was quantified by real-time qPCR; relative number of rRNA gene copies distribution was calculated for all twelve collected fractions, but only presented here for the fractions for which T-RFLP analysis was performed

SIP-experiment with ^13^C_10_-naphthalene and ^12^C_10_-naphthalene as a control was performed to investigate whether *Clostridia* or *Desulfobacterales* play an active role in naphthalene degradation. DNA was harvested after 72 and 97 days of incubation resulting in early and late labelling. The time difference between the sampling was caused by different growth rates in the biological replicates (Fig. 2b). After density centrifugation, the distribution of bacterial 16S rRNA gene copies in ^12^C- and ^13^C-gradients was investigated in six fractions which retrieved approximately 97% of the loaded DNA (Fig. 8). T-RFLP analysis was performed on the six gradient fractions with most of the DNA detected. In both samples (late and early labeling, Fig. 8a, c), the “heavy” fractions at 1.701 and 1.703 g ml^−1^, respectively, were dominated by the 149 and 215 bp T-RF peaks of *Clostridia* and were shifted from the “light gradients” with the highest *Clostridia* peak by 0.008 g ml^−1^ (corresponding to 20% label incorporation). According to (Lueders 2017), 20% labeling is regarded as the detection limit for SIP. In contrast, the 160 bp T-RF peak of the *Desulfobulbacterales* was dominant at 1.696 and 1.694 g ml^−1^ in the late and early labeling experiments, respectively (Fig. 8a, c). T-RF peaks of *Clostridia* and *Desulfobulbacterales* did not show any shifts between the collected fractions in control experiments where ^12^C-naphthalene was used as a carbon source (Fig. 8b, d). Therefore, the incorporation of ^13^C-naphthalene is reflected in the DNA of *Clostridia,* suggesting that these are the key-degraders of naphthalene.

To verify the results from the SIP experiment, the metagenome of culture 1MN grown on ^13^C-labeled naphthalene from day 97 underwent metagenomic sequencing. Raw sequencing reads assembled into 171 scaffolds, longer than 1000 base pairs. To identify the organism involved in naphthalene, 1-, and 2-methylnaphthalene degradation, we screened for fumarate addition and naphthalene carboxylase genes previously reported to be involved in PAHs degradation (Kleemann and Meckenstock, 2011; Mouttaki et al. 2012). Only two putative fumarate addition genes encoding proteins with low identity (31 and 39.5%) to the naphthyl-2-methylsuccinate synthase (nmsA) gene of the sulfate-reducing enrichment culture N47 were identified in the entire metagenomic dataset (Figure S4, Table S2). These genes were located on 131- and 127-kb long scaffolds, respectively. The scaffolds encoded for 121 and 128 other proteins, respectively, 106 and 105 of which were classified as *Clostridia* (103/101). Four genes on the scaffolds were annotated as ribosomal proteins from members of the *Clostridia*. To identify genes encoding for naphthalene carboxylases, the metagenome was screened by blastp using sequences of the previously described putative naphthalene carboxylation gene cluster of the naphthalene degrading culture N47 (Bergmann et al. 2011). A whole gene cluster was detected on a 51-kb long scaffold coding for 47 proteins of which 39 have blast hits against the Uniref100 database. 21 of the proteins were annotated to proteins of *Clostridia*. Three of the proteins have blast hits against four putative naphthalene carboxylases in N47 with gene identities between 32 and 51% (Figure S5, Table S3, Table S4). The presence of the putative nmsA genes and the putative naphthalene carboxylase gene cluster on scaffolds classified as *Clostridia* further supports that the *Thermoanaerobacteraceae* are the key-degraders of 1-methylnaphthalene in the enrichment culture.

## Discussion

The present study describes a culture which was enriched with 1-methylnaphthalene as electron donor and ferrihydrite as electron acceptor. Despite our efforts of performing serial dilutions to extinction we could not obtain a pure culture, so far.

### Phylogenetic affiliation

T-RFLP analysis, pyrosequencing data and phylogenetic analysis showed that the enrichment culture consisted mainly of two types of microorganisms belonging to the *Thermoanaerobacteraceae* (approximately 60% of community) and the *Desulfobulbaceae* (approximately 29% of community). Stable isotope probing revealed that the biodegradation of naphthalene was carried out by members of the *Clostridia.* This was also supported by the detection of a putative naphthalene carboxylation gene cluster on a scaffold classified as *Clostridia* (Figure S5, Table S3, Table S4). *Clostridia* species in this study have 85% classification reliability to the order *Thermoanaerobacteriales* and the closest relatives (92% similarity) in the gene bank are clones from an enrichment with oily sludge under mesophilic and thermophilic conditions (Cheng et al. 2014). Moreover, there is only 88% similarity to members of the *Peptococcaceae* identified in an enrichment which also degrades naphthalene and 1-, and 2-methylnaphthalene under iron-reducing conditions (Kleemann and Meckenstock 2011). The closest described cultivated representatives are a hydrogenogenic, carboxydotrophic, thermophilic marine bacterium *Calderihabitans maritimus* KKC1(T) (Yoneda et al. 2013) (88% sequence identity), *Moorella perchloratireducens* An10 (87% sequence identity) (Pierce et al. 2008), and *Moorella thermoacetica* AMP (86% sequence identity) (Balk et al. 2008). *C. maritimus* KKC1(T) and *Moorella* species are spore-forming thermophilic bacteria, which can use thiosulfate, fumarate, Fe(III), nitrate or perchlorate (Pierce et al. 2008) as electron acceptors and produce acetate via the Wood-Ljundal pathway (Tindall et al. 2010). *C. maritimus* KKC1(T), in particular, is able to produce H_2_S from CO oxidation coupled to sulfite or thiosulfate reduction (Tindall et al. 2010). The very low similarity on 16S rRNA sequence level of the clostridial member in our enrichment 1MN suggests a new species if not a novel genus. Nevertheless, this awaits isolation and strain description.

The closest relatives of the *Desulfobulbaceae* in the gene bank (99% sequence identity) were clones isolated from an aquifer sediment column amended with acetate under sulfate-reducing conditions (Handley et al. 2013), from an industrial site contaminated with petroleum hydrocarbons (Zhang et al. 2012b), from an uranium-contaminated groundwater where acetate was used for bioremediation (Elifantz et al. 2010; Holmes et al. 2007), and recently discovered cable bacteria in groundwater aquifers (98% sequence identity) (Müller et al. 2016). The closest described relative (88% sequence identity) is *Desulfurivibrio alkaliphilus* AHT2 (Melton et al. 2016). Also for the *Desulfobulbaceae*, the phylogenetic distance of more than 5% from their closest described neighbors suggests that these two organisms might represent novel genera (Tindall et al. 2010).

### The role of gram-positive microorganisms in the degradation of aromatic hydrocarbons

Several studies have demonstrated that gram-positive bacteria play an important role in the biodegradation of aromatic pollutants such as biphenyl by *Desulfotomaculum* (Selesi and Meckenstock 2009), benzene by *Peptococcaceae* (Kunapuli et al. 2007), and *Pelotomaculum* (Abu Laban et al. 2009), or naphthalene, 1- and 2-methylnaphthalene by *Peptococcaceae* (Kleemann and Meckenstock 2011). Only few pure gram-positive strains belonging to the genus *Desulfitobacterium* (Villemur et al. 2006; Kunapuli et al. 2010), *Desulfosporosinus* (Robertson et al. 2001; Liu et al. 2004) and *Desulfotomaculum* (Plugge et al. 2002; Morasch et al. 2004) capable of degrading monoaromatic hydrocarbons under iron- or sulfate-reducing conditions have been described. Although an enrichment culture dominated by one microorganism belonging to *Peptococcaceae* capable of naphthalene degradation coupled to Fe(OH)_3_ reduction has been reported (Kleemann and Meckenstock 2011), no pure anaerobic strains of gram-positive PAH-degrading bacteria have been described so far. The difficulty in isolating such strains indicates the importance of the co-cultured members reported here. Recently, *Desulfobacterales* have been shown to play an important role in hydrogen consumption (Burow et al. 2014) in the environment. Similarly, the *Clostridia* in our enrichment might produce hydrogen during consumption of naphthalene which in turn would be consumed by *Desulfobacterales* pulling the reaction towards complete naphthalene oxidation to CO_2_. However, also other types of interaction such as a cryptic sulfur cycle are possible.

The current enrichment culture is the second culture reported to degrade naphthalene, 1-methylnaphthalene and 2-methylnaphthalene under iron-reducing conditions and the first to degrade PAHs in a community consisting of *Thermoanaerobacteraceae* and *Desulfobulbaceae* with Fe(OH)_3_ as an electron acceptor. It is not clear, why there was no sulfate reduction observed as *Desulfobulbaceae* are also expected to reduce sulfate. This example demonstrates that Fe(III)-dependent biodegradation of PAHs might play an important role in iron-rich, anoxic habitats. *Thermoanaerobacteraceae* are prominent community members in oil reservoirs (Canganella and Wiegel 2014; Cheng et al. 2014) but to our knowledge were not shown to degrade hydrocarbons, so far. Our culture reveals that the *Thermoanaerobacteraceae* can be involved in hydrocarbon degradation but the exact mode of their electron accepting process remains open.

### How *Thermoanaerobacteraceae* can interact with *Desulfobulbaceae*?

The current study does not provide direct evidence on relationship of *Clostridia* and *Desulfobulbaceae* in the 1-methylnaphthalene degrading enrichment, but gives some suggestions on their possible interactions. One option could be a synthrophy. Based on SIP analysis, Kunapuli and colleagues (Kunapuli et al. 2007) proposed that under iron-reducing conditions gram-positive members of culture BF metabolize benzene and produce H_2_, while gram-negative *Desulfobulbaceae* consume H_2_ and transfer electrons to Fe(OH)_3_. To our knowledge, no degradation of naphthalene by synthrophic communities rather than methanogenic has been described so far (Gieg et al. 2014). On another side, our experiments where the 1MN enrichment culture produced sulfate and ferrous iron in the absence of any carbon substrate (and this was coupled to increased abundance of *Desulfobulbaceae*) might suggest that *Desulfobulbaceae* can grow independently from *Clostridia*. Active degradation of naphthalene (and AQDS reduction) always started after a lag phase where up to 2 mM of Fe(II) has been produced. We suggest that *Desulfobulbaceae* oxidize or disproportionate elemental sulfur coupled to iron reduction. In the absence of organic carbon sources, the *Desulfobulbaceae* are probably capable of CO_2_ fixation to build up biomass. As soon as their biomass is high enough, *Clostridia* can start degrading 1-methylnaphthalene and excrete electrons in the form of easily degradable substrates/metabolites which can be further oxidized by *Desulfobulbaceae* and coupled to rapid Fe(II) reduction. Thus, 3,4-dihydroxybutanoic acid has been detected in culture supernatants (Supplementary material, Fig. S1C) and could be one of the metabolites excreted by *Clostridia* and consumed by *Desulfobulbaceae*. Follow-up metagenomics analysis will give more detailed insights into functional capabilities of *Clostridia* and *Desulfobulbaceae* from our enrichment culture.

### Degradation of 1-methylnaphthalene

Several studies exist on anaerobic degradation of naphthalene or 2-methylnaphthalene under sulfate-reducing conditions in sediments (Coates et al. 1996; Anderson and Lovley 1999), enriched microcosms (Bedessem et al. 1997; Sullivan et al. 2001; Zhang et al. 2003) as well as in sediment-free liquid cultures (Annweiler et al. 2000; Meckenstock et al. 2000; Musat et al. 2009; Kümmel et al. 2015). However, the described sulfate-reducing cultures are able to degrade only naphthalene and 2-methylnaphthalene but not 1-methylnaphthalene. In fact, there has been only one study so far that reported on a culture oxidizing 1-methylnaphthalene coupled to iron reduction in sediment-free highly enriched culture (Kleemann and Meckenstock 2011).

In sulfate-reducing microorganisms, 2-naphthoic acid is a central metabolite produced either via carboxylation of naphthalene (Zhang and Young 1997, Meckenstock et al. 2000) or via addition of fumarate to 2-methylnaphthalene and subsequent beta-oxidation (Meckenstock et al. 2016) (Fig. 1b, c). Detection of 1-naphthoic acid as a metabolite during the 1-methylnaphthalene biodegradation in our study indicates that 1-naphthoic acid can be another central metabolite in anaerobic degradation of naphthalenes besides 2-naphthoic acid (Fig. 1a). Most likely, it is produced via fumarate addition to naphthyl-1-methylsuccinic acid, similar to the degradation of 2-methylnaphthalene. This was supported by detection of putative fumurate addition genes encoding proteins analogues to naphthyl-2-methylsuccinate synthase (NmsA). The low identity of corresponding genes (Fig. S4) to NmsA from sulfate-reducing enrichment N47 might indicated a new clade of fumarate addition genes in the *Thermoanaerobacter*-related microorganisms. Further degradation steps in the downstream metabolism of 1-naphthoic acid including ring reduction and cleavage as well as beta-oxidation of dicarboxylic acids are probably also similar to the degradation of 2-naphthoic acid. However, the specific metabolites must be different up to the step where ring I of the 1-naphthoic acid is cleaved by a hydrolase reaction and the first acetyl-CoA unit has been removed by beta-oxidation leading to a cyclohexane ring with one acetyl and one carboxylic acid side chain.

## Electronic supplementary material

Below is the link to the electronic supplementary material.

Supplementary material 1 (DOCX 710 kb)
