# Supplementary material for: Anaerobic degradation of 1-methylnaphthalene by a member of the Thermoanaerobacteraceae contained in an iron-reducing enrichment culture
Source: Biodegradation. 2017 Nov 24;29(1):23–39. doi: 10.1007/s10532-017-9811-z (PMC5773621; doi:10.1007/s10532-017-9811-z)
Supplement: Supplementary file 1 — Supplementary material 1 (DOCX 710 kb) [file 10532_2017_9811_MOESM1_ESM.docx]

**Supplementary material**

**Fig. S1.** Growth of culture 1MN with 30 mM Fe(OH)_3_ as electron acceptor in medium reduced with 0.7 mM Na_2_S in the absence of an organic carbon source. A) Time series of Fe(II) (circles) and SO_4_^2-^ (triangles) in active incubations (filled symbols) compared to abiotic controls (open symbols). Error bars represent standard deviations of two biological replicates. B) Electropherogram of FAM-labelled 16S rRNA gene amplicons from DNA extracts of one replicate culture shown in A) digested with the restriction enzyme MSPI.

**Fig. S2 (A)** total ion chromatogram of metabolites (derivatized with BSTFA) extracted from culture 1-MN cultivated with 1-methylnaphthalene dissolved in HMN. Mass spectra profiles of **(B)** 1-naphthoic acid; **(C)** 3,4-dihydroxybutanoic acid; and **(D)** benzophenone.


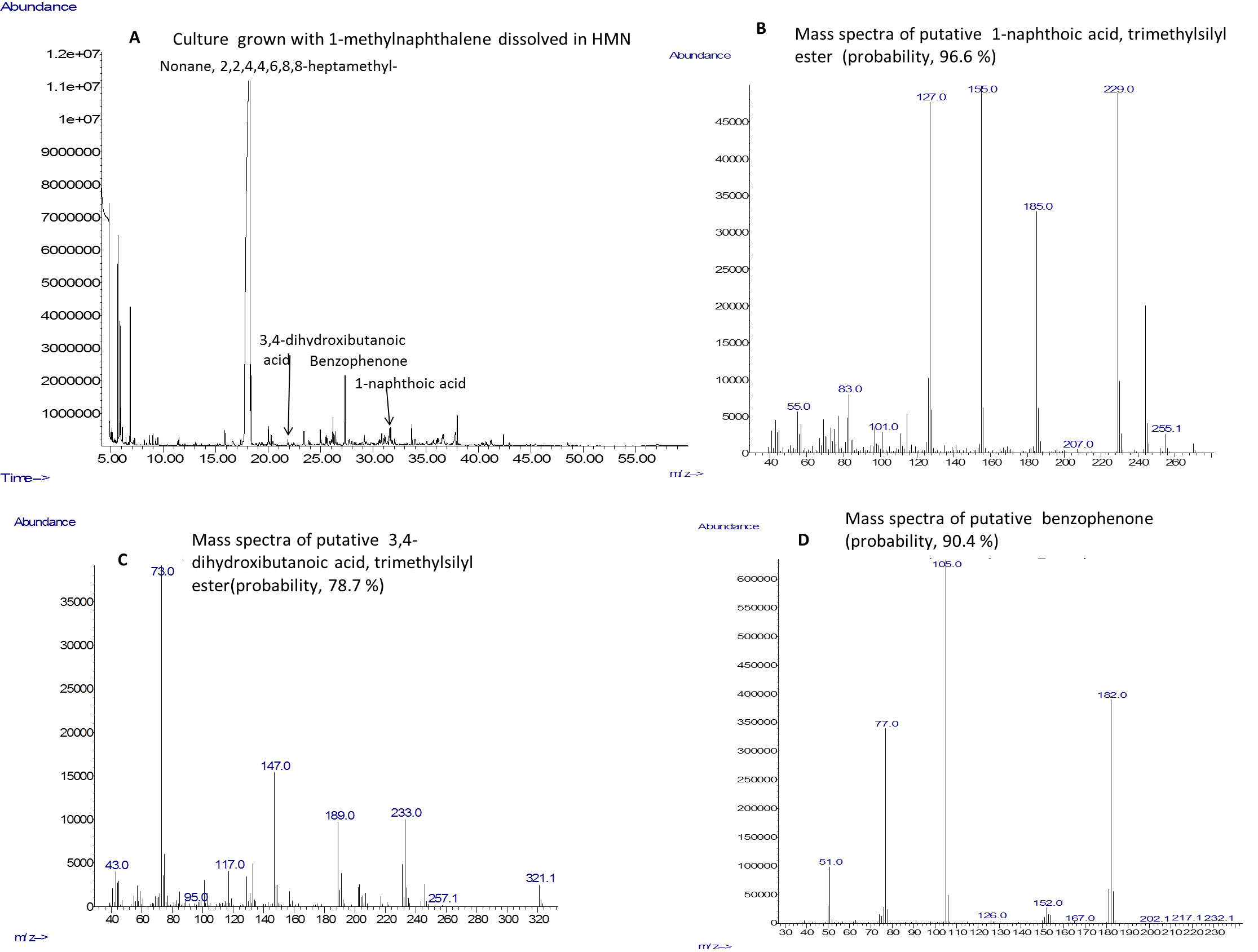


**Fig. S3.** Bacterial 16S rRNA gene T-RFLP fingerprint of enrichment culture 1MN cultivated with naphthalene (upper panel), 2-methylnaphthalene (middle panel), and 1-methylnaphthalene (lower panel) expressed as relative abundance, measured as a percentage of total peak height of a given restriction fragment. Time of culture cultivation is indicated in brackets.


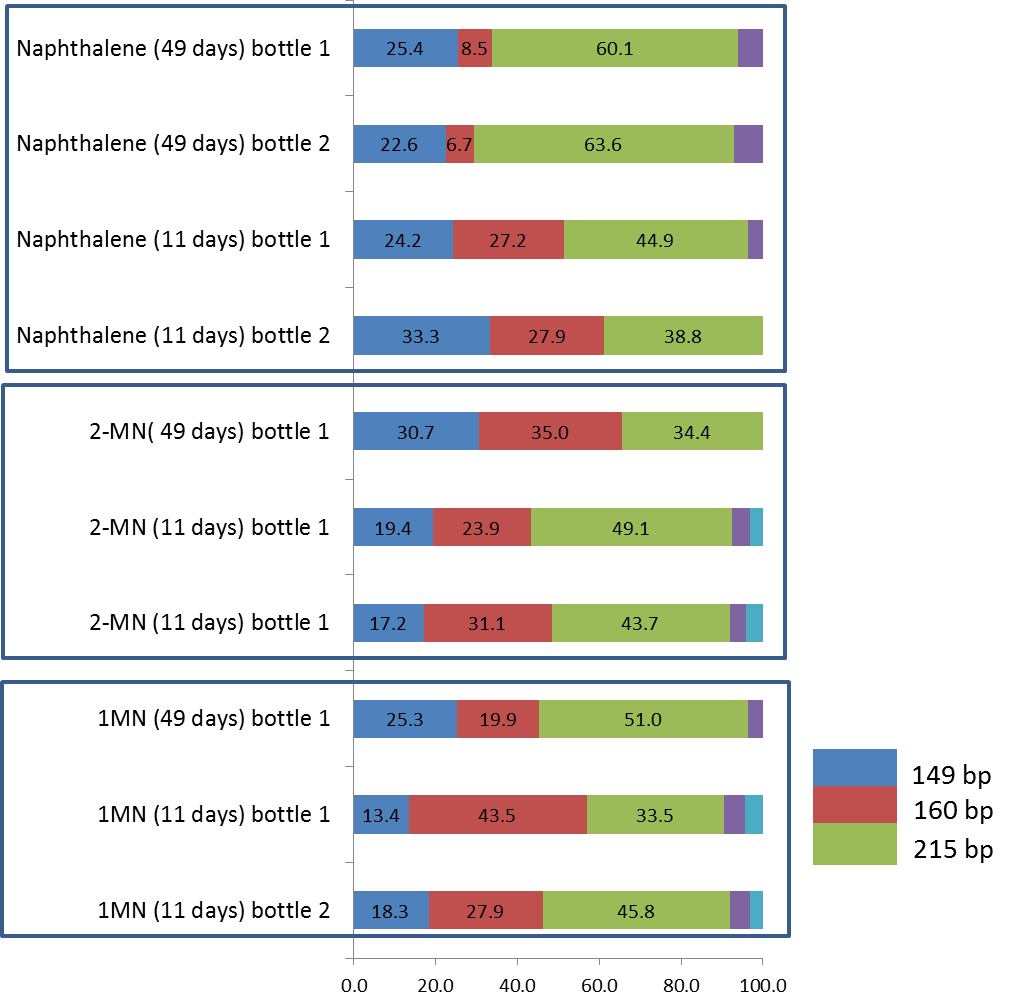


Relative abundance, %

**Supplementary Table S1.**

Specifications of assembled 16S rRNA gene contigs from amplicon sequencing via Seqman II software

**Assembled 16S rRNA gene sequences from amplicon sequencing of enrichment 1MN**

>Seq1

TACTCTCGTGAGAGTTTGATCMTGGCTCAGGACGAACGCTGGCGGCGTGCCTAACACATGCAAGTCGCACGGTTCTGGGGGCTGGAAGCTAGGGGCTAGAGGCTGGAAAAAGGGCAGGTATTGGCAAAGCCGATGCCAACGAGGAATCCAGCATCAAG

CATCCRGCTTCCAGCTTCCAGGGCAGTGGCGGACGGGTGAGTAACACGTGGATAACCTACCCTTCAGACCGGGATAACCCTGGGAAACCGGGGCTAATACCGGATACGTTCCTGTTGGGGCATCCTGACGGGAAGGAAGGCGGCCTCTGGGAAACCAA

GCTGTCGCTGAAGGATGGGTCCGCGGCCCATTAGCTAGTTGGTGAGGTAAAGGCTCACCAAGGCGACGATGGGTAGCCGGCCTGAGAGGGTGGCCGGCCACACTGGGACTGAGATACGGCCCAGACTCCTACGGGAGGCAGCAGTGGGGAATCTTGCG

CAATGGGGGAAACCCTGACGCAGCGACGCCGCGTGGGTGAAGAAGGCCTTCGGGTTGTAAAGCCCTGTCATAGGGGACGAAGTCTGTAGGGTGAATAGCGCTACAGGTGACGGTACTCTAAGAGGAAGCCCCGGCTAACTACGTGCCAGCMGCCGCGG

TAATACACGAGAGTA

>Seq3

TACTCTCGTGAGAGTTTGATCCTGGCTCAGGACGAACGCTGGCGGCGTGCCTAACACATGCAAGTCGCACGGTTCTGGG

GGCTGGAAGCTAGGGGCTAGAGGCTGGAAAAAGGGCAGGTATTGGCAAAGCCGATGCCAACGAGGAATCCAGCATCAAG

CATCCGGCTTCCAGCTTCCAGGGCAGTGGCGGACGGGTGAGTAACACGTGGATAACCTACCCTTCAGACCGGGATAACC

CTGGGAAACCcGGGGCTAATACCcGGATACGTTCCTGTTGGGGCATCCTGACGGGAaGgAAGGCGGCcTCTGGGAACCA

GCTGTCGCTGAAGGATGGGTCCGcGGCCCATTAGCTAGTTGGTGAGGTAAAGGCTCACCAAGGCGAcGaTGGGTAGCCG

GCCTGAGAGGGTGGCCGGCCACACTGGGACTGAGATACGGCCCAGACTCCTACGGGAGGCAGCAGTGGGGAATCTTGCG

CAATGGGGGAAACCCTGACGCAGCGACGCCGCGTGGGTGAAGAAGGCCTTCGGGTTGTAAAGCCCTGTCATAGGGGACG

AAGTCTGTAGGGTGAATAGCGCTACAGGTGACGGTACTCTAAGAGGAAGCCCCYGGCTAACTACGTGCCAGCMGCCGCG

GTAATACACGAGAGTA

>Seq4

TACTCTCGTGAGARTTTGATCCTGGCTCAGGACGAACGCTGGCGGCGTGCCTAACACATGCAAGTCGCACGGTTCTGGG

GGCTGGAAGCTAGGGGCTAGAGGCTGGAAAAAGGGCAGGTATTGGCAAAGCCGATGCCAACGAGGAATCCAGCATCAAG

CATCCGGCTTCCAGCTTCCAGGGCAGTGGCGGACGGGTGAGTAACACGTGGATAACCTACCCTTCAGACCcGGGATAAC

CCcTGGGAAAaCCcGGGGCTAATACCGGATACGTTCCTGTTGGGGCATCCTGACGGGAAGGAAGGCGGCCTCTGGGAAA

CCAAGCTGTCGCTGAAGGATGGGTCCGCGGCCCATTAGCTAGTTGGTGAGGTAAAGGCTCACCAAGGCGACGATGGGTA

GCCGGCCTGAGAGGGTGGCCGGCCACACTGGGACTGAGATACGGCCCAGACTCCTACGGGAGGCAGCAGTGGGGAATCT

TGCGCAATGGGGGAAACCCTGACGCAGCGACGCCGCGTGGGTGAAGAAGGCCTTCGGGTTGTAAAGCCCTGTCATAGGG

GACGAAGTCTGTAGGGTGAATAGCGCTACAGGTGACGGTACTCTAAGAGGAAGCCCCGGCTAACTACGTGCCAGCCGCC

GCGGTAATACACGAGAGTA

>Seq7

TACTCTCGTGAGAGTTTGATCCTGGCTCAGATTGAACGCTGGCGGCGTGCTTAACACATGCAAGTCGAACGAGAAAGGG

ACTTCGGTCCTGAGTAGAGTGGCGCACGGGTGAGTAACGCGTAGGTAATCTACCTCTGCATCTGGGATAACACTTCGAA

AGGGGTGCTAATACCGGATACACTTATGGATCGCAAGGTCTATAAGGAAAGGAGACGATCCGCAAGGAGTTTCTGTGTA

GAGATGAGCCTGCGTCCCATTAGCTAGTTGGTAGGGTAAAGGCCTACCAAGGCGACGATGGGTAGCGGGTCTGAGAGGA

TGATCCGCCACACTGGAACTGAAACACGGACCAGACTCCTACGGGAGGCAGCAGTGAGGAATATTGGACAATGGGCGCA

AGCCTGATCCAGCGACGCCGCGTGAGTGATGAAGGCCTTTGGGTCGTAAAGCTCTGTCAGCAGGGAAGAACGGCCGTGT

GGTTAATACCCATGCGGATTGACGGTACCTGCAGAGGAAGCACCGGCTAACTCCGTGCCAGCMGCCGCGGTAATACACG

AGAGTA

**Figure S4:** Neighbor-Joining dendrogram of putatitve NmsA sequences from 1MN culture grown on ^13^C_10_- naphthalene after 97 days of incubations. NmsA-like sequences (labelled with solid circle) were aligned with the AssA/BssA/NmsA sequences of reference strains. The dendrogram was rooted with pyruvate formate lyase (PFL) as outgroup. The accession numbers are given next to representative sequences. Abbreviations: Ass; alkylsuccinate synthase, Mas; methylalkylsuccinate synthase, Bss; benzylsuccinate synthase, and Nms; naphthylmethylsuccinate synthase. The scale bar represents 10% sequence divergence. Bootstrap test (1000 replicates) is shown next to the branches. The scale bar represents 10% sequence divergence.


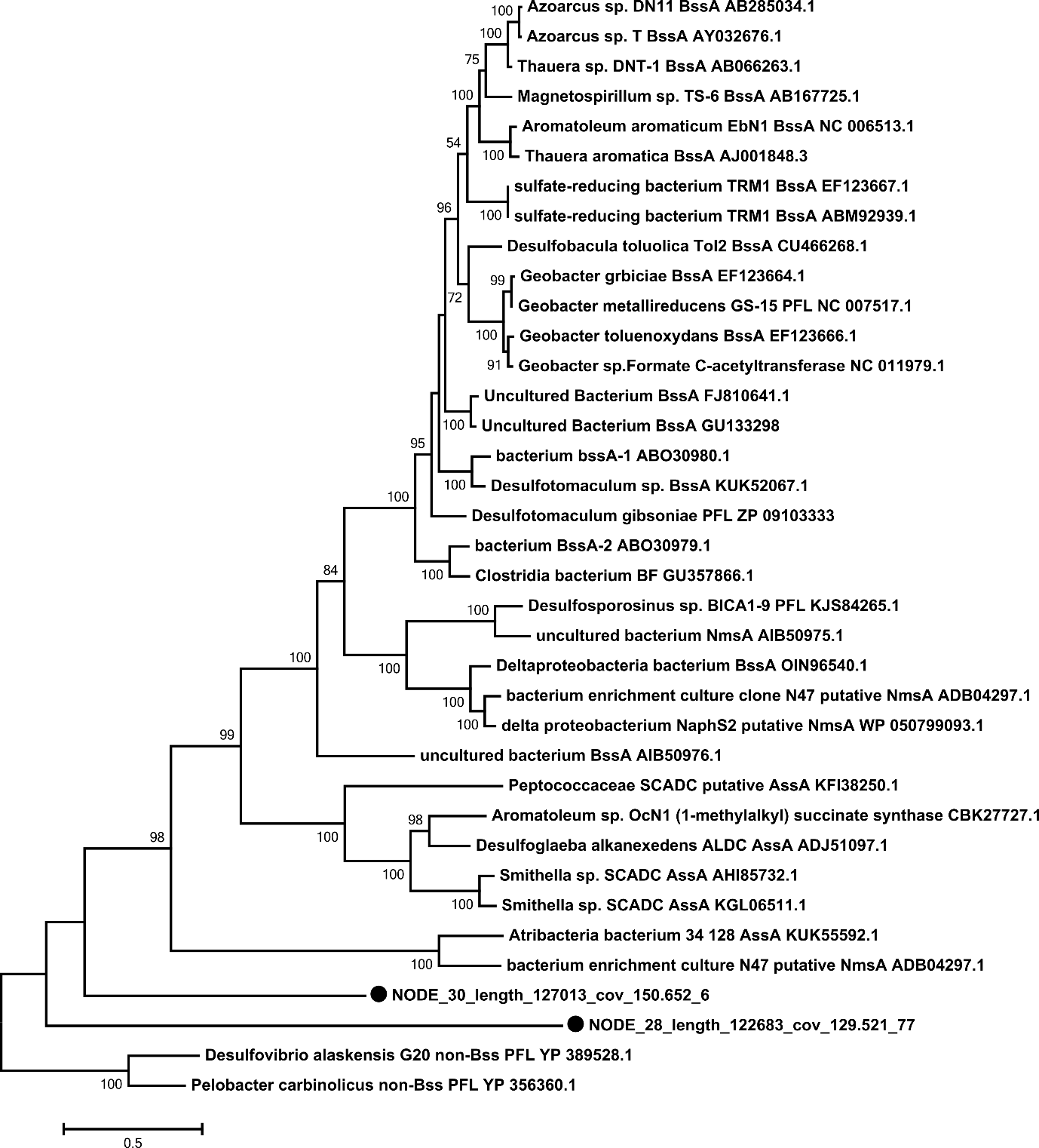


**Table S2.**

**Sequences of two putative fumarate addition genes encoding proteins**

>NODE_28_length_131Kb_cov_148.143_77; putative fumarate-adding enzyme

MTTPDFKPGPWQEEINVRDFIITNYTPYLGDSSFLTGPTPRTSKLWNKCLELLQEEHRRGGVYKVDARTPITITSHPPGYIDRDLEIIVGLQTDEPLKRAVNPFGGLRMARSACAEFGEDVDPEVCRIFTEYRTTHNDGVFAVYTDEMLALRRSGVITGLPDAYGRGRIIGDYRRVPLYGVDRLIAAKVADIRELPVLQRITRDTIQLREEIRQQVNSLEDLKKMAAGYGFDLSRPAADARDAIQWLYLAYLGAIKQQNGAAMSLGRVSTFLDIYLERDLHAGRLTEEQAQQLIDDFVIKLRLTRHLRTRSYNELFAGDPNWVTEALGGMALDGRPLVTRTTYRFLQTLYNLGPAPEPNMTVLWAADLPRPFKEYCARVSIDTSAVQYENDDLMRPIFGDDYAIACCVSAMRLGKQMQFFGARCNVPKALLLTLNQGVDELTGEKIAPVFYLPRGREEVLDYREVWPAFRRVLDWLASKYVEVMNIIHYMHDKYAYESLQMALHDSLVGRFMAFGLAGLSVTADSLAAMKYARVRARRDDRGIAREFSIEGAYPAFGNNDDRVDRLAVELVQTFMEALRRYPTYRNAVHTLSVLTITSNVVYGHKTGATPDGRQAGEPFAPGANPMHGRDRKGALAALSSVAKIPYQDALDGISYTFSITPRALGPDPQARVDNLIALLDGYFKQGGHHKNVNVFDREILQEAMEHPEKYPQLTIRVSGYAVHFIKLSREQQEEVIARTIF*

>NODE_30_length_127013_cov_150.652_6; putative fumarate-adding enzyme

MSIAEVKLGEMRLSFQYGKVPKEVTDREIRKEPSKRAKKLRDDYLNAKISLDIEFPYWYTRKWIEEEGQHPLIRRALALKCGFEHLTPMIRAGELLVMQKTRYIRGAFVMPWTANRYPLSIEERMEHEAEEASKMSLEEVVVLAKGGGNVTQSAGNVLSISGRFGIRREEFPMLVEVCRYWKNKSSEDTCFMWAAMHPKYDQYLNMKKAVLMHVDLEYSLRHGRNVVNYQLPLQIGFKGMIDACTEKINANIKEGNADKIAFWKATIIVIEGVQAWIRNYAKEAKRLAAKEKNVKQKQEFEEIAGRLEWIAENPPRTFIEALQLCWTCHIAVVNELQISGLSPGRLGQVLYPFWKQDIKEGRITREQTLEILECMRVKFTEIEIAQSVGTIGLTGGSTFNNLCIGGVNPDGTSAENELEELIIESAMTCATPQPTLTVLYDGKLSEKFLLKAIECNKIGTGYPAWVNNRVAMEYLMKTFKDEGITLEDARAWTIGGCLEIQPGALVNGRLGAGSYSSTGVGFINMPKVLELVLWDGVDPRTKVRVFPPHGSKLETYEELYRAWQQYFYEVVTVFEEMYNLKAAAMFNIDNPIFYSALMADCIEKGLDMDRGGCRYNRTLTTWITGQVNLANSLASIKKNVYEERNFTLDELKNALINNFGYRSALETSQFSLLDQKRETDEWARIHSLCVNAPKYGNDDPYVDEIYKDVIEYWRDVVPQVKDVFGRPWVPCQLSVASHGPLGQACIASADGRLAGLTLADAAQSPYPGTDLNGPYAVLNSAVIIDHSDYQNTQLNLKVHPSCIKGTQGSRKLLELIKAYMDKGGYHIQFNVVDTRMLRDAQDHPENYRDLLVRVAGFTAYWVELSKPIQDEIIARTEYGEI

**Figure S5**

**(A)** Maximum likelihood tree of putative naphthalene carboxylases in 1MN (filled symbols) and N47 (open symbols), and related UbiD family carboxylases selected from NCBI´s non-redundant protein database. The tree represents a consensus tree of 100 bootstrap replicates. The percentage of replicate trees in which the associated taxa clustered together in the bootstrap test are shown next to the branches. (**B)** Gene organisation of 1MN genes in comparison to respective blastp hits (same shading) within the naphthalene carboxylase gene cluster in culture N47. The symbols represent the corresponding sequences in (A).


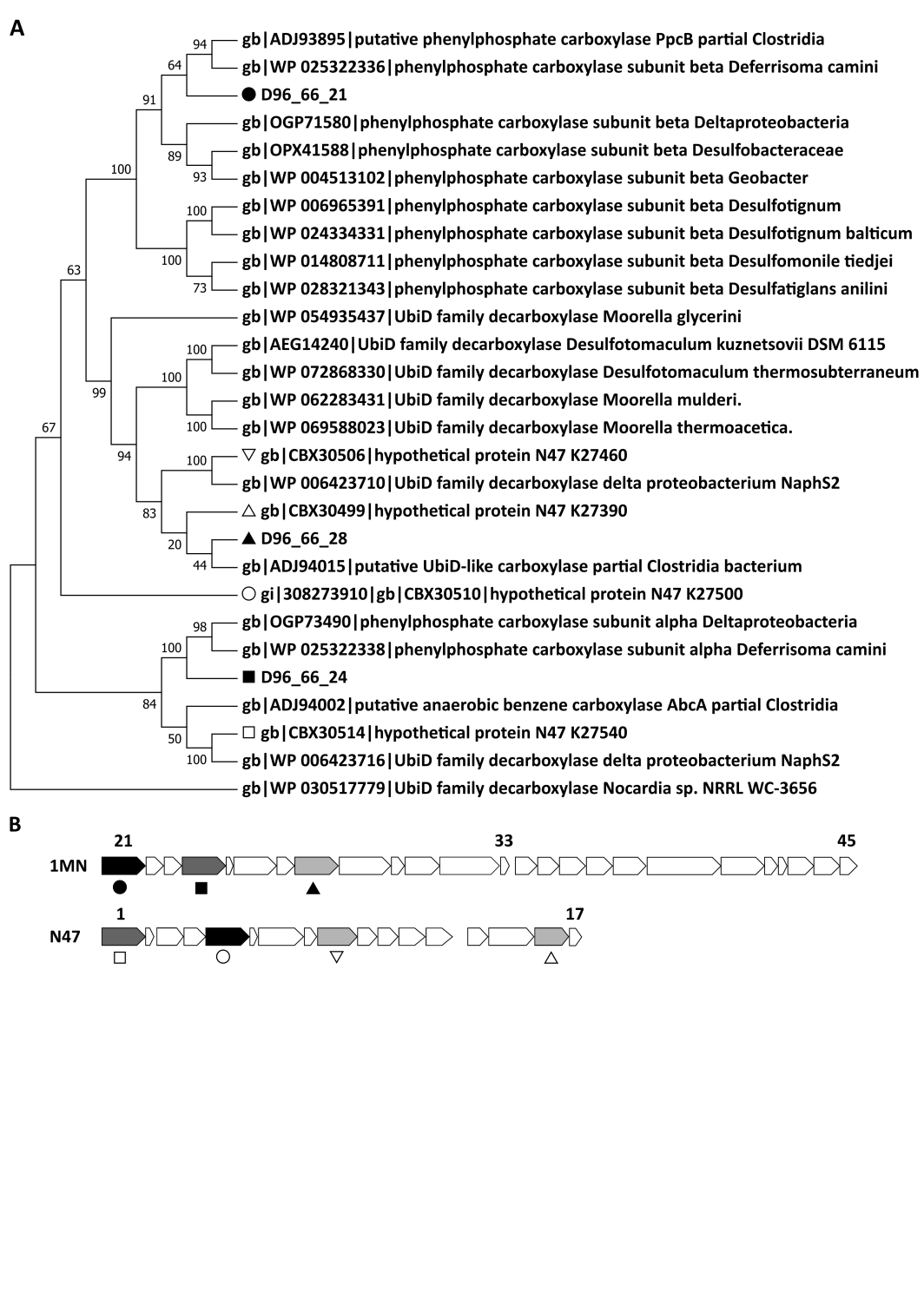


**Table S3.** Gene annotations of scaffold 66 (excerpt) and blastp hits against putative naphthalene carboxylases in enrichment culture N47

| **ORF** | **Uniref_100** | **Length (aa)** | **Identity (%)** | **Bit-score** | **Annotation** | **Organism** | **Hits in putative naphthalene carboxylation cluster of culture N47** | | |
| --- | --- | --- | --- | --- | --- | --- | --- | --- | --- |
|  |  |  |  |  |  |  | NCBI (nr) | Identity (%) | Bit-score |
| 21 | A0A1F9BPN9 | 494 | 55.5 | 545.8 | Phenylphosphate carboxylase subunit beta | Deltaproteobacteria bacterium | CBX30510 | 32 | 138 |
| 22 | A0A1F9BT12 | 186 | 73.5 | 295.4 | Uncharacterized protein | Deltaproteobacteria bacterium |  |  |  |
| 23 | A0A1F9BVE3 | 184 | 77.9 | 274.2 | Uncharacterized protein | Deltaproteobacteria bacterium |  |  |  |
| 24 | UPI00046D15DA | 478 | 61.5 | 622.1 | Phenylphosphate carboxylase subunit alpha | Deferrisoma camini | CBX30514 | 51 | 465 |
| 25 | A0A1F9BV94 | 83 | 45.2 | 69.7 | Uncharacterized protein | Deltaproteobacteria bacterium |  |  |  |
| 26 | A0A0U2L5W7 | 464 | 43.8 | 388.7 | Phenylacetate-coenzyme A ligase | Paenibacillus naphthalenovorans |  |  |  |
| 27 | D8WWD5 | 202 | 70.5 | 268.9 | Putative UbiX-like carboxylase subunit | Clostridia bacterium, culture BF |  |  |  |
| 28 | A0A0K1J7B3 | 454 | 43.9 | 350.5 | UbiD-like carboxylase subunit | Azoarcus sp. CIB | CBX30506; CBX30499 | 36; 42 | 303, 318 |
| 29 | A0A1F8TBK2 | 631 | 47.9 | 584.3 | Aldehyde ferredoxin oxidoreductase | Chloroflexi bacterium |  |  |  |
| 30 | A0A1F9BZJ5 | 143 | 54.5 | 148.7 | Uncharacterized protein | Deltaproteobacteria bacterium |  |  |  |
| 32 | A0A1M5B1M8 | 402 | 35.3 | 211.1 | Glycosyltransferase | Desulfotomaculum australicum |  |  |  |
| 33 | A0A0M2U3X0 | 732 | 41.0 | 436.0 | Uncharacterized protein | Clostridiales bacterium |  |  |  |
| 34 | UPI0005550B90 | 126 | 73.6 | 198.4 | S-adenosylmethionine decarboxylase proenzyme | Clostridiales bacterium |  |  |  |
| 35 | A0A0M2TZA5 | 242 | 35.5 | 134.0 | Uncharacterized protein | Clostridiales bacterium |  |  |  |
| 36 | F6DRR2 | 272 | 51.1 | 243.8 | Demethylmenaquinone methyltransferase | Desulfotomaculum ruminis |  |  |  |
| 37 | A0A1F8RSF7 | 337 | 47.1 | 318.9 | Uncharacterized protein | Chloroflexi bacterium |  |  |  |
| 38 | A0A0M2U4A7 | 313 | 63.6 | 378.6 | UTP--glucose-1-phosphate uridylyltransferase | Clostridiales bacterium |  |  |  |
| 39 | D3FBQ7 | 388 | 51.2 | 357.5 | Putative glutamate--cysteine ligase 2 | Conexibacter woesei |  |  |  |
| 40 | A0A0M2U3G3 | 842 | 39.4 | 508.1 | Uncharacterized protein | Clostridiales bacterium |  |  |  |
| 41 | A0A101ECR7 | 572 | 73.4 | 865.1 | Fumarate reductase, flavoprotein subunit | Moorella sp. |  |  |  |
| 42 | A0A0T6ARF9 | 144 | 53.4 | 166.4 | Uncharacterized protein | Armatimonadetes bacterium |  |  |  |
| 43 | A0A0M2U3S6 | 121 | 51.7 | 117.1 | Uncharacterized protein | Clostridiales bacterium |  |  |  |
| 44 | A0A1F8M755 | 287 | 61.7 | 365.2 | Succinate dehydrogenase iron-sulfur subunit | Chloroflexi bacterium |  |  |  |
| 45 | A0A1J4WUH9 | 276 | 51.6 | 272.3 | Short-chain dehydrogenase | Parcubacteria group |  |  |  |
| 46 | UPI0004712E7E | 210 | 45.2 | 185.3 | Transcriptional regulator | Caldicoprobacter oshimai |  |  |  |

**Table S4. Scaffold of sequences of putative naphthalene carboxylase.**

>NODE_66_length_50651_cov_165.733_1

MPILMSLILVSYGIAASVLVSVTRGNGNASLGAWFTAAGMFVTIGVMVTGELLKRIKSQNDKL*

>NODE_66_length_50651_cov_165.733_2 UniRef100_M0C2K4 Na+/solute symporter Taxonomy=Archaea; Euryarchaeota; Halobacteria; Natrialbales; Natrialbaceae; Haloterrigena

MMTTVGIISTAFIILYVIIGIWSARTARKSIDEYLAAGRRIGMLVGGFALYAAFCSAMVYVGLPGMTYSLGLNATWQITLGAALGFGLLYFLLAEPVRRFAQFTLVDYLAARYNHLAIRILVALFICGIFTFAIVPQIIGAGLMIGNTFGVSLRTGEIIFAVVLLLYVSIGGMRAVTFSDAFQGIIMFIPLMVVGLLAIAYFGGITPMIAQAAQNNPKIVEPTLPLVSYLGLVFGIALGQSSAAHIIMRISSAASPPVARTTVVVGVVLAVLMYFLASNILGIAAWAVSPGLSKPDNALWAVMEKIIPQWLFGWIIAGLVAAAISTASAMLMGSVASLVSDIYYRVLNPNASPGELYWRGIVITWALGVAAVLVSLFASQQVSWFVAIQSQALGATFFFPLVLGIWWKRTTTAGAISGMVGGLGSFILFTALKILPLYGPVVPAIIISLCLTVLISLLTQPPPVEVVEACCTKLHQRE*

> NODE_66_length_50651_cov_165.733_3 UniRef100_A0A0M2U9E6 Uncharacterized protein Taxonomy=Bacteria; Firmicutes; Clostridia; Clostridiales

MDGEGTQLLFGRDFTEAELALVREVVALFPRLSSKELAATLCEHLGWATSAGRLKVESCLKLLLKLEAKGEIKLLPKARKAKRKESAPLLSKRTDPPSQELAGELWEFEPVWVLVQDKEGMWLWNEYIQRYHPLGYQRPFGAHQRYFIVCGGGELLGCISFAASAWAVEARDRWIGWSQSDRSRRLHLVANNNRFLIFPWVKVKNLASRVLALVVRRIREDWQARYGYGPVLLETFVDLEYACTCYRAANWIKLGQTAGRGRMDRDTQYLSTPKRIYVYPLVREFRAVLCGKEKGGSG*

> NODE_66_length_50651_cov_165.733_4 UniRef100_A0A0M2U7Z0 Uncharacterized protein Taxonomy=Bacteria; Firmicutes; Clostridia; Clostridiales

MKESLLLNRRLMWRELRAKQKEKGIKPRPRASLTNGKAGWQTVEEERAGRWQVVEECHKVWRVVLPVLLKRLAKIPDPRNPRSSKHKLQRPEKPLTFPGWERNSGTRIGLSCGMRIYWLVPGPAGAE*

>NODE_66_length_50651_cov_165.733_5

MPLNIPEAAAPVDLRLAGPDSPCRFLSPRPAILPTFAPWSRTPVPLRRSSCRVHGTSETEIRNCAQLNSCTPPRFPPPPQTWKPSRHKASITGKSYLFRKHCVHRHAPRQEEALPSLPPPPAQRSRAMGLLSAPRTPCIAVVALALGSHAGAQPGRPLKGPASRTPSAAGTCPQRGPLLRAAVPLFFHPGP*

>NODE_66_length_50651_cov_165.733_6

MGRQCFYRQKIVTQARSVEAKTLVSYLVGEIRSRRELAPEEAALVAEDALEYLMHLADRGPGQIDFPAILGLDAHWGAPGGTSPSR*

>NODE_66_length_50651_cov_165.733_7

MLEEFGVVAMQVGRMARCIEEAYFQGCLLDWPRLGVLFPYNHQALRQRLEPLFGRREHSCPWRA*

>NODE_66_length_50651_cov_165.733_8 UniRef100_A0A0C7NNL1 Uncharacterized protein Taxonomy=Bacteria; Firmicutes; Clostridia; Thermoanaerobacterales; Thermoanaerobacteraceae; Moorella_group; Moorella

MELYLRGEDLTSVRKKLCCSRTAWRRWWQLFCQVGGLGEEDPEEAARRLGQPVVLVRGFAELWAEVRENTRLSEKVKREGLWALAAPPGGGPRQAFRQRLLERHRYTPAAADQFEQELRELASRLSSRGRSAGQIVWTGVAQDQPPAAAWETAAWFPPYWTTWCRGLGRKEYK*

>NODE_66_length_50651_cov_165.733_9 UniRef100_A0A174LI01 4-hydroxyphenylacetate decarboxylase activating enzyme Taxonomy=Bacteria; Firmicutes; Clostridia; Clostridiales; Lachnospiraceae

MSSSQERALIFDIQGFSVHDGPGGRTLVFFKGCPLRCYWCCNPEGQNQYREVMYRRSKCQMCYRCMDSCPNGAIHVKNKGDFIAIDRSKCGYCNNLDCVKGCYNGALKVVGKYMTIDELMMKIERDRRFWGTGGGVTLGGGEVMLQYKFAARLLEECHASYIHTAIETSGYAPWSHYQEVLKNVDWLFVDIKHMDPDIHRKGTGVSNVSNLENIERMASHQKDYQMVIRIPVIPGYNDNTENIVATAKFMKRIGVHVINILPFHRLATSKYEQLDRKYACKNMESPRLGDMYQIQNIFHNYGLMCYVGSDTPF*

> NODE_66_length_50651_cov_165.733_10 UniRef100_K4LX65 Transposase, IS4 family Taxonomy=Bacteria; Firmicutes; Clostridia; Thermoanaerobacterales; Thermoanaerobacteraceae; Thermacetogenium

MYIRTIQRKNKDGSVVRYVQLAQNEWDPQARCAKARVLYSFGREEEVDREALKRLIRSINRFLGPEEMLRYEAETTGQNLLRFLSSRPLGGAWALNQLWEELGIKEVLLKLLKKRQFKAPVERAIFAMVANRALDPTSKRGVQEWVEEDVVIPDLDQIPLQQLYRAMDFLLENEAEMQKQVYYALANLLNLQVDLLYFDTTSVYFETEEEDEDGLRRRGHSKDHRPDLPQAVIGLAVTRDGLPVRCWVWPGNTADMSVVEQVKKDLIGWKLGRVITVLDRGFNSEDNLRYLQRAGGHYIVGEKMRSGRENTVKALSRPGRFQTVRDNLEVKEIVVGEGEARERYILVRNPQEAQRDKAKREAVIKELEEQLPKIRSHAKAVCELMAHPVYGRYLKLDSRGLPKIDRAKIKEEEKLDGKYLLRTSDDTLSSEDVALGYKQLLLVEDAFRTLKSRLEPRPVYHRLEDRIRAHVLLCWLALLLIRVAENKTGQTWKNLRSTLERMHLGEFSGECGQVW*

>NODE_66_length_50651_cov_165.733_11

MTISVHMATMALVLWLKQCSKFQFCFKSLKQLFSMSHLRWAIRQITSALVLDLGAVVTR*

> NODE_66_length_50651_cov_165.733_12 UniRef100_A0A0B7MER2 Uncharacterized protein Taxonomy=Bacteria; Firmicutes; Clostridia; Thermoanaerobacterales; Thermoanaerobacterales_Family_III._Incertae_Sedis; Syntrophaceticus

MAGRTKKYAEPGRLDPKNDFIFRKLFSSPGNEDLLTDLLNSILQPTPEKTICQVNILNPIKPRDHRADKEAVMDILARTNDGTLVTIEIQVADEHNMAQRAVYYWSVVFTSQMVTGMTYAEIKKTISINILDYVFLKQTARYHTVFHLCEETEGFQLTDVEEIRFVDLPKMLDKWRKSELIRETEPLTKWFLLLEANEDQEIAKELEAMAMSDAALQKAIREWERLSRDPETIAQYISRMKGKMDRLSALKTAEERGMAKGRAEGMAEGRAEGRAEAKQEAICTLLDKRFQPVAGELQEKVRQMTAEKVLDRIYEELLEAETVEQAKQIISRAGKTKPR*

>NODE_66_length_50651_cov_165.733_13 UniRef100_UPI00083023C5 UPI00083023C5 related cluster Taxonomy=unknown

MPDIFVEVVPDLGARWVDKSFCYRVPPVLKEKVAPGCLVQVPLGRRMVYGFVVRRVEEPPAVAIKDIAGVSGLRPLGPDLLDLARWMGRRYFYPLGAVLGMMVPSPAREGTGRQRRYAVLDVPAEAAAAEAERFQARAPAQARVLRGLVTANGLAMEELGPARRSLAALVSRGLVRVEDYPVYREGYSGVEEMADRAGPPALSAEQQEAVARIMGAVEGAGFAPFLLHGVAASGKTEVNLRLVEEVIRRGRHCLFLVPEIALVPQVLAQLRRRLGSEHVVVWHSSLSPGQRHDAWEMAASGRAGVVVGTRSAIFAPVPRLGLVVLDEEQEPAYKEDQGCRYHAREVALKRCQLARAVLVMGSATPSLETYSRARSGVYRLVRLQQRVTGTGPPRICLVDMREEVKAGNTGILSRLLVRRLQEVLEAGRQALVFINRRGFYPYVYCGNCRFVWRCPHCDVGLTYHRAREELRCHYCGYALPYPQPCPACGATRVWQGRGAGTQQVEAELTRLFPAVEVLRVDLDTTRSSW*

>NODE_66_length_50651_cov_165.733_14 UniRef100_UPI0005CAF0E5 hypothetical protein Taxonomy=Bacteria; Firmicutes; Bacilli; Bacillales; Bacillaceae; Bacillus

MTEPIQGVIFVNPEVRDDLFYWQAARRYPWDEFTDIAQQFLQARYQELKRRLPQVEGQAELFKDAVDLDSSCGSGERKSPRGRKGIPFWPLFRAFSLARLMRVEDSARDVYFLLKNNPTFARACGFEAVPSYRVTARFDHIMTHHGLWAKARIKAVQLNLDKQVFFPLRGNSSGYHPRRGRGNSSSKG*

> NODE_66_length_50651_cov_165.733_15 UniRef100_F7Z2V8 Transposase IS4 family protein Taxonomy=Bacteria; Firmicutes; Bacilli; Bacillales; Bacillaceae; Bacillus

MDTTHVEAEATAPPKDKKDDPAYQHTDDNVGVLRKSNTVTYIAHKVALVVDANEDFCYTHCTFKGNTSDPETLEGTLLKFKEEFPEVAKEVEIVLADGIYQSANNQKVSKEVLEAKLYAPINPRNRKSVKLENVRGITEIDPYGRPKCLSGRCLDLVGRDQKQQQYIWGCPVFGIRHQETLDCPEANHLQCCNLNAGGRYYRTNRTDFPQIDWENPQHSVRFGLHYNREVPLNG*

> NODE_66_length_50651_cov_165.733_16 UniRef100_A0A1M5ACZ3 Transposase IS116/IS110/IS902 family protein Taxonomy=Bacteria; Firmicutes; Clostridia; Clostridiales; Peptococcaceae; Desulfotomaculum

MQDILEICCGLDVHKETVVACLLKGDTDGEPKTTIRIFSTLLAGLDELRAWLEAENCRHVAMESTGVYWQPVYSVLEEAFDGSMVLTVTNARHMKNVPGKKTDMKDAEWIATLLRAGLLQGSFIPVKPIRELRNLTRYRKSIMEEITAQKNRIEKHMQSCGFKLSTFLTDIFGVSGRAIMDHLCRHGKISAWEVDALVKGRAKSKLHEIKQAVNGKMDLHQREFLKLLLVWLDQHYEHLRQVEQKLEEKMAQYQRQLEQLDGIPGIDKTAAAAILAEIGIDMSHFKTAEHICSWAGLSPGNNESAGKKVHSHHQW*

> NODE_66_length_50651_cov_165.733_17 UniRef100_Q39TT7 Metal-dependent phosphohydrolase Taxonomy=Bacteria; Proteobacteria; Deltaproteobacteria; Desulfuromonadales; Geobacteraceae; Geobacter

MQRAKIREVYSLARPFLNTRHNDIHTVISYGYALRLLRDEGGDRETVIPAILLHDVGWSSVPEHLQLTAFGPNPTNPALNRVHEVEGARLARDILRQATYDEQKIEEIAEIIVGHDSRLEALSLNDMVVKDADKLYRFSRWGFAIDVKRFRRDPVVHLQWLGQQIDRWFFTARAMALARGRILAEQSDNLF*

>NODE_66_length_50651_cov_165.733_18

MLMLSAKEDHAQTLISRDMIEAIDRVRTIVGVQVAGVALYDAAQVVFE*

> NODE_66_length_50651_cov_165.733_19 UniRef100_A0A1F9DPQ6 Uncharacterized protein Taxonomy=Bacteria; Proteobacteria; Deltaproteobacteria

MTEKLIYWLEELGVEDNDLVGKKCANLGELTKAGVRVPPGFALAVQAYERFLATTGAREEMQRFLETFKADPNDPKDLAKFEKASEVLREIVDSKEMPSDMADIISAHYDKLCQRTGISDVPVAVRSAGPVSRPGQYETYLWVIGQKDLMKKIIRVWSSTFNHRSMVYRAQNGLPMEFDPIGCAVIQMVDAKVSGVMFTLHPANGDVSKIVIEANWGLGESVVSGRLSPDFYVVDKQTLNIVEKRVSDKTYEYLLDPATGATDFFEVPQERRNIPCLTDEELVELAKLGLFVEKHYGKPQDTEWTISKDLPFPQNIFMVQSRPVTAKMSKKKTATDTILDMMMSRLYQL*

> NODE_66_length_50651_cov_165.733_20 UniRef100_UPI0005597D8B hypothetical protein Taxonomy=Bacteria; Proteobacteria; Deltaproteobacteria; Desulfobacterales; Desulfobacteraceae; Desulfatiglans

MPYGYYHYDWRGEPDLDPQYYDCWVCNVAHSVPAWTPLFTSEWYRLIPYAMQYANDYICEPTSKGWDWRIYNGYAYITSIKVPESEVEGRKQVYREQLASIIDDPWGYSERLFSRLMEKYDYYKPLNVKEMRDCELVQHLWDMAELDRLMWEVHMLGWDGLLGGLRLWREMLVDLFGITPYDVRYAKLLSGFDNALFKLNSDLTHVAARAVELNVESNFSLPDEEVIPAMEQTEAGREWLGEFDKFLNEHGYRSDRMLEFTRPTWLEKPTLAIPDIRRIISAGVSHAPDILRHQLRKEGEEIERELLAKLPSDQREWFHKLTQCAQAAHSWSESHDYWCEFQTYGLRRRAIKELSERLYRKDIIDDPEDTAYLLHQDLIYAAVIQEKVGKKYFRDLIKRNKEEFEYYKSIPPGGDTTPLFLGDPSKLPLVAGRDTLFGVVAAPPAEDAAKVGAVAVGCAGAPGIVEGTARVIASASEWDQIKPGDILVCPMTDATWTPLFALLKGVVTDSGGLLAHPAIVCREYGIPAVCGTFDASKKIKTGDRIRVDGNLLRVYKLDQ*

> NODE_66_length_50651_cov_165.733_21 UniRef100_A0A1F9BPN9 Phenylphosphate carboxylase subunit beta Taxonomy=Bacteria; Proteobacteria; Deltaproteobacteria_**putative_naphthalene_carboxylase**

MSTVETATDQKTMGGMVLMKPIRNLRDYIEVCEAEGEVKRVKAEVDWDLELSHIAKINEEQGGPALLFENVKGCLGSVLIGVFSSPKRMAIALGMPPEYTMCQMADEWRRIGSRKPIPSVEVNTGPIMENIVEEKDIDLTKFPAPKYFPLDGGRFMGTSAFRITRDPETGELEIGMGRMQLYDEKNVGLYLSPGRGGDKIRAKYEKLGKPAPFALVFGCDPALALASVMFIKGASKYDIAGTLRGIPVETVVSDFTGLPIPAEAELVVEGFMDHDDLRPEGPFGDVTGLYTSELQKPIPKRFVKAKRVLYRNDPIMLANSSGRPVSDVQMMISLPRTAALWQELEAMKIPGIKSVYIPPQMAGRFWAIVSVKTQYPGHANQVAHAVISTTTGHLGIKGVIVVDDDIAADDMDRVLWALATRYLPDRDTEIIKRGRSSPLDPAVPPETGYVEIISKILIDATVPYEWPQKPVVAALDEEVVKKVKSRWKEYGLD*

> NODE_66_length_50651_cov_165.733_22 UniRef100_A0A1F9BT12 Uncharacterized protein Taxonomy=Bacteria; Proteobacteria; Deltaproteobacteria

MGLKKDRETAVEKATKVKLFAHDVHGVLTSSAFFCDIEGRRQYSFWHMDGFGDLSLSANGIRIAFLDTTSVDGEGLYRAKELKLDQYYYKVSDKVAKIRELEQELGISDENVGYIGSEITDAAAMRICGFRVATADAVDEIKELADYVTTAPGGRGAIREVCEFILGSMGLWEAWTEKVMRMGYK*

> NODE_66_length_50651_cov_165.733_23 UniRef100_A0A1F9BVE3 Uncharacterized protein Taxonomy=Bacteria; Proteobacteria; Deltaproteobacteria

MGNGKSREAAVEKAKRTKFVILDIHGVLTDNTLYYTDDGKKSERFSLRDRLGCLALMEGGISVAFLTSKISRADEQVGKIYNIPTEYLWGSSAKMARLDEFEKDSGLKDEDFCYVGDEMIDLGIMKRVGFSVAPADAASEVKEIADYISSAGGGQGVVRELAEFILTAQGKWEAIKEKISASG*

> NODE_66_length_50651_cov_165.733_24 UniRef100_UPI00046D15DA phenylphosphate carboxylase subunit alpha Taxonomy=Bacteria; Proteobacteria; Deltaproteobacteria; Deferrisoma_**putative_naphthalene_carboxylase**

MAFRDNREFFEALLRTGDGIRVAREVDWDLEMGAIVRRVCEMGGPAPLFESIRDYPGWRALGAPLATFRRLAVALGLPAHASVREISEVYIEKTRAPGPEPKLVSTGPCKENVVVGDEVDLFMLPAPMVHEGDGGRYLSTWHMVVSKDPDTAEVNWGMYRQMVIDEHTMVGPLLPVSDTGRVFHNKYKPKNKPMPFATVIGADPTSSIAAAAGVPWQEPVFASALSGEPVELVKCETVDLEVPATAEVVLEGEILPGKELPEGPFGEYPGYRTSLREPRTVYRVNAITYRNNPILPGANMGVPVDEGQLLRAFTLGLEARRILASQGLPVSEVFMPPCSAHHLMIVSVKSAVTNIAVQVAHALFGSKLAPWFYYVVVVDADVDIYNLEEVIHAICTGCHPGRGIRIYEDDIGSFATPFLSLEERRQGRGAKAVFDCTFPPDWDPKTELPIKVSFETNYPKEVKERVIANWKDYGFPQ*

> NODE_66_length_50651_cov_165.733_25 UniRef100_A0A1F9BV94 Uncharacterized protein Taxonomy=Bacteria; Proteobacteria; Deltaproteobacteria

MEFEIYVSRLEELPVEQECELLIRALTPSDRRKKYKYQKVRALISQQKDRYPDLLWVRFLKGQLHTDPYSIRIANTKGATDE*

> NODE_66_length_50651_cov_165.733_26 UniRef100_A0A0U2L5W7 Phenylacetate-coenzyme A ligase Taxonomy=Bacteria; Firmicutes; Bacilli; Bacillales; Paenibacillaceae; Paenibacillus

MFCDFASPKAVTMTREELREYHLYHLQRLLERVYANSAFYRKLWANAGLHPEHIKTLDDFTRLVPMVTKQDFLADQNSSPPYGQRLAVSEEEICQVHLTSGTSGIGQEVYALTRDDISLEAQGWVIHLRSIGLKPGDVSIVTWPVATMAAALNVYEACREIGVNAFLVGIYDGETKIKLMQRFDMNHLVATPTYVSRLAVLCQEMGLNPSQAFGNLKAISVAAESYPANWLEEMEAVWGCPIHDVYGSTQNATALGMTCQTRRSAGEGGGTRRNMHLFEHLTFVEAIDYESGEPVSYGAEGELVLTPLFRQATPVIRFRTGDKVILYPGQTCSCGSQFDFLEAGTIARFDDMIKIKAANVWPQTVDDIIFSYKEVEEYNGVVSIDEKGRENVRVLLEFRDGVPEGTKASLMKQVGERIRQKTQVTMIVEEVPRGTLERTLFKSRRWVDRRQEGLRQVVRYLEK*

> NODE_66_length_50651_cov_165.733_27 UniRef100_D8WWD5 Putative UbiX-like carboxylase subunit (Fragment) Taxonomy=Bacteria; Firmicutes; Clostridia; environmental_samples

MTARLIVGMSGATGSIYGIRLLEVLRDLGVETHLVMSRSAEKNISLETKWRVEDVRALATVVYPFEDIAAAISSGSFLTCGMVVAPCSIRTLSGIANSYNENLLVRAADVTLKERRRLVLLVRETPLHTGHLRLMTEVTEIGGIILPPMPAFYHHPQTIDDIINQTVGKALDMFGIEHHLFCRWSGGEAAAGVVQGPAGER*

> NODE_66_length_50651_cov_165.733_28 UniRef100_A0A0K1J7B3 UbiD-like carboxylase subunit encoded in anaerobic phenol operon Taxonomy=Bacteria; Proteobacteria; Betaproteobacteria; Rhodocyclales; Rhodocyclaceae; Azoarcus_**putative_naphthalene_carboxylase**

MDMRAYLDLLQDAGEIMVIEDELDPVYEASAVLGTREVQMGPAVLFRNLKGYPGWEVLGNLFGSRRRVALYLGVHEDEAEAAFVRRQQELKPPVRVKDGPCKEVRVREPIDLLRELPILTFSEGDVAPYITAGIMVSHDPHTEERGVSVHRLRVAGPNRLGTMLMNPPLNLFMQKAEKAGTPLEVAICIGPEPAVLLATLTRWTPGIDKYEIAGGIAGSSLEVVRCETVNIEVPAASEVVIEGRILPNVREPDGPFGEHSGCYVVSNEGRIVEVTAITRRQHPIYQVIKPWTTELDLLMALGQGNSTLRRLKEVVPEVEAIHVVPLSSMLAAVIKLSSTSRARVRKAILTCLAIDPRIKQVVAVDDDIDITNPYEVAWAMVTRFQADRDVVILRDTEGTPLDPSRKAGNLTSKIGFDATSADMEGNFKKISPPATAVIRAQEVVGQYLRRYSS*

> NODE_66_length_50651_cov_165.733_29 UniRef100_A0A1F8TBK2 Aldehyde ferredoxin oxidoreductase Taxonomy=Bacteria; Chloroflexi

MPLSRQLLFVDLSSGNSQSVPIPLEVRQGFLGGRGLNMLLLYRLGLRCHDPFSSDNPLIFGAGLLTGVLGGRLNISARSPESGLLGDSNVGGDFGAELAATGYSHLVIVGRSDRPVYLIVTEDGVEIRDAGYLWGRDTLETQRMIRRELTDPEVRVACIGPAGENLVRFAGVVTGQKNIAGRTGMGAVMGSKNLKAVAVKGSRGFRISDPDGYLEAVKDVVGQVSSTRWGQALGKMGTPLLLHYSNAMGFLSVRYHQRTTLGEQGRLLEPSALEEFSTGMLACYGCPVHCRHRYAIDAGSRYAGAAGEGPEYASVASLGSHVGNLNIEAVIYMAQLCNRYGLDTISTGNYLAWAMDLYERGILTSADTSVPLEWGNEDSLIQMLHMIANRRGFGNVLAEGSRAAAELGPEAAKHLLQIKGLSMELTDERPVKSFALGLATATRGCCHMRSRPSVDVTNLPRNILAGLYGGDVGRDYTEYEGKGRMVWWHELFHAVTDAIGYCRFLSIFSSIHAVGYQEYAKLIHRATGLELDAGELQEIGERIYTTERMFLTQQGISRKDDTLPDLYFDVPVPAGPSRGKLISRTRFQEMLDEYYGLHGWNEDGIPRPETVSRLRLEEFLGGGWLAYPGS*

> NODE_66_length_50651_cov_165.733_30 UniRef100_A0A1F9BZJ5 Uncharacterized protein Taxonomy=Bacteria; Proteobacteria; Deltaproteobacteria

MGVGGLRIRVASSEVCSGCRLCEIVCSLYHLGEVNPRLAAIHVVKDDLGTSMNNPRVCLQCKDKTCLKGEEVDEKAEMSAFIWPVGRAQKCPYGALHVHNGQAYHCDLCGGDPRCVQVCTTGAIAVAGKEDGHGKVREREGS*

>NODE_66_length_50651_cov_165.733_31

MGKSGNEKVVKVHLPGHLAGLAGDRRVVAGHGDTVEEVLASVREQFPGLSEALGGAGMASFSIYINDAAVEAREGLRTKVRDGDDIYLVVPLAGG*

> NODE_66_length_50651_cov_165.733_32 UniRef100_A0A1M5B1M8 Glycosyltransferase involved in cell wall bisynthesis Taxonomy=Bacteria; Firmicutes; Clostridia; Clostridiales; Peptococcaceae; Desulfotomaculum

MARRRVLLLVRPAVGGIATHVLSLALGLDRQEFSPVLVAPEGYGVLERAREQGLASYGVRFGDGLNPWQDRKAVARIREIAVREGTGLVHSHGLKADLLASLATRTGTIKHIATVHTFPVRRAGLTGFASRFLTRYVSGRVGHYIAVSQALAAELTGRYGVDPEKVSVVPNGLAREKLEDYSRQKLVRPDPAMDGPVIGSVGRLVLEKGMEDFIRAARLLRGEFPQARFWIVGDGPLRARLQALVSRLGLEGKVSLLGYQAEVAPWLAAMDVFVTCPVSEGFSLVTLEAMASGKPVVATATGGLPELIRSGVNGLLVPVRDPAALARAVSLLLRRPDVASELAVKARQHVCQRYTAESMAKQTQAVYERVLRQDSSGGGLVGFGVCAPSRTASSWGGEDRA*

> NODE_66_length_50651_cov_165.733_33 UniRef100_A0A0M2U3X0 Uncharacterized protein Taxonomy=Bacteria; Firmicutes; Clostridia; Clostridiales

MKVWVAAAVLAIVSVGFSRPALAQSPSGRVVVVVVADRMSLADLAGADQPWPELLSRAGIALMNTNTGGGRTPENALATISAGGPAVAAPDAALAFRADEGFAGEEAGLTFTRRTGRTAAPENVVVLAWPQIAGNNRQRDGNPQPGFLGETIRRAGGKTAVLGNADMPDSPRREAALLAMDTGGIVDYGLPGKDTVTTTSQDRLLPLVTDYDRLRQAYRQLPGDANLVVIEVGDMARLDRLDSLGTAEVVAREKQTIMRDIGYFLQKLLADEPARVIFLVPTPSSPAAAEKNLLTPLLLFARDVPPASWLTSPSTRRPGLVSNVDVTATILSWLGQPLPPGLAGRPVAPAPATPPRPVEALLDFNERSVFVYALRPPLLKTYVGAQIVVIAGAVLGLLAGCSRLLGWWRPVLLALVSVPLALLLLPLLPSYGSPIFYTGIFLLLTALLTVAALRFSRSTLAAFGLLTAATWTSLIVDVFTGSNLGRQSILGYDPMAGARYYGIGNEFMGVLVGAAAMTIGIGLQLHRGREPLSSWPLAGVAVILGLTAAILAAPQYGANAGGTIAILIAACFLWAKLARGGLDWSRVALAGVAVFAVLIGMSLADARRAVEVQSHFGRTWELIGSEGVGPILDIAARKAAMNLKLIRYTIWSRVLLVALGTLVLLCYRPVNLWRHVWRSNPHFYNGFSTAVVGAVAALIFNDSGIVSAATVMTMAMAPFLYLLLQARAGPE*

> NODE_66_length_50651_cov_165.733_34 UniRef100_UPI0005550B90 S-adenosylmethionine decarboxylase proenzyme Taxonomy=Bacteria; Firmicutes; Clostridia; Clostridiales

MHALGRHVLAEIYGCEFGILNDEKKVEEIMVNAALAAGADVRESIFHKFSPQGVSGVVVISESHLAVHTWPEYGYAAVDVFTCGEKVSPWEACRYVASELGAAHLNATEVHRGLFADPHQKAVNL*

> NODE_66_length_50651_cov_165.733_35 UniRef100_A0A0M2TZA5 Uncharacterized protein Taxonomy=Bacteria; Firmicutes; Clostridia; Clostridiales

MLDILQPVQSELRLVELRMRKEFALKEGRLNELLPLHLKNPWDRLFLPAMVLLHNKLFAPIKEKTVAMACVFQFIYLATLTHRQIETNPAMVVLVGDYLYTKFFSYLCRHEALEYLDPLSRSICQIQEAGIWRQQQRSVQTDSDNLDIIDKERALLVSQACGCGAELGGAGEEQIQMSRGFGLHIGRMWALSEAEPGPRTGVERAEALKCLYRLPGGEARETLERLLLHLDSFAETVSVVG*

> NODE_66_length_50651_cov_165.733_36 UniRef100_F6DRR2 Demethylmenaquinone methyltransferase Taxonomy=Bacteria; Firmicutes; Clostridia; Clostridiales; Peptococcaceae; Desulfotomaculum

MAASDGGGGSLVAPAVDKGQMVHEVFATIARQYDRMNSLLSFGRDKGWRQRAVDILAPGAADTVLDVCCGTAMLSLEVAKRLGPQGKVIGIDFSPEMLAVGERNLRANPLGSRVELVPGDALELPFPDDSFDSAVSAFALRNLTDVVAAMAEMRRVVRPGGRIVILELAKPSATVFQRIYYFYFYHLVPLIGRVAVGRSLPYSWLPESVRIFPSQAEVQAMLSMAGLVNTSYQDLTLGTAAIYWGTKPERPRGLMEAGGDSPQAARSNTTS*

> NODE_66_length_50651_cov_165.733_37 UniRef100_A0A1F8RSF7 Uncharacterized protein Taxonomy=Bacteria; Chloroflexi

MPRLEPTEITPHVYQCPIPIPNNPLENLLAYLVQTPAGTWMIDAGWNTPEAIAAWESHLPALNLSFRDITQIIITHLHPDHYGLAGTIRERSGAPVVMSRIEAEQISSRYQDYRQLLAEVGSWLSRNGVGQGELPDLQQASLRVLNRVGPVQPDATLREGDLLEAGFTTLQVIETPGHSPGHICLFAREAGLLFAGDHVLPTISPHIGMYPQSPPNPLAVFFHSLDKVDGLKVNLVLPAHEYVFDNLRQRICQIKEHHRIRLEEILAALGDEEKTAYRVAAHISWAIGPWEQFDVWARRSALMEVVAHLEYLLGQGRVAKRCRPDLVLYRRLEVRK*

> NODE_66_length_50651_cov_165.733_38 UniRef100_A0A0M2U4A7 UTP--glucose-1-phosphate uridylyltransferase Taxonomy=Bacteria; Firmicutes; Clostridia; Clostridiales

MKQVRKAVIPAAGLGTRFLPATKAQPKEMLPVVDKPAIQYIVEEAVACGLEDILIITGRNKRAIEDHFDHSLELEAALAEKDNHEALENLRRISEMADIHYIRQKEPRGLGHAIYCARKFIGQDPFAVLLADDLIVTDGSPCLQQLLDIYETRPGNIIAIQQVLPEEVNKYGIVEGQALLPRLYQVSRLVEKPPVGSVSSNLAIVGRYILQPEIFTALAEVAPGAGGEIQLTDAIGRLLGPQPVYAYLFQGRRFDVGDKLGFLQATIEMALMRPDLRDELGAYLRGTFMTDSRLAEVVYNGGSGGFSGGQEL*

> NODE_66_length_50651_cov_165.733_39 UniRef100_D3FBQ7 Putative glutamate--cysteine ligase 2 Taxonomy=Bacteria; Actinobacteria; Thermoleophilia; Solirubrobacterales; Conexibacteraceae; Conexibacter

MEAPEAFLEGRSFENGQDLTIGLEEELQILDPRTLHLTNKFGELKKAVPADLDPWVKGELIASEIEIATVRSQDFSSAAGDFLSKHRQLVELALAHDLYLGATGVHPFSPWYDQEIIDTPHYRLVEGELRYVAWRNNTFSFHVHIGVKGRERAILVCDALRTFLPHLLALSASSPFYEGRYTYLHSVRTQLFAKNFPRCGLPDAFGSWASYRRYLEFLYRSNSIHEDTQIWWSIRPHLQWGTVEVRICDAQPWPEDTLKIASLVVALAARVLLAVDRGEPLPVYPRCYLEENFWRATRYGLGGLLVDLETEREVPAVEAIRGLLAWTRPVHDELCLKPYLEPLGEFMAAGNWARRQIRAYEAGHDLVAIHRYLAGLTMGLAVAGTCG*

> NODE_66_length_50651_cov_165.733_40 UniRef100_A0A0M2U3G3 Uncharacterized protein Taxonomy=Bacteria; Firmicutes; Clostridia; Clostridiales

MSGKQRKQGRPEPRAGGSGAARHQTSKSNKTSTPERGKGAARPHSGGLPSRGSGGAGVRADGREKPVLPAGSPGRAWYWAAFTGVSVLLFYPPYFRGLFFQPEQLWTLLLAAGVFALVWGWKISTRDLRWLTHPFDFLALALVLVYTAATLVAANQHLAMAEVVKVALYFLVFWMVARLSQVRGQRDNLLAVLYASAAGVALAGLLTATGFIAIKDGFVAGRIYSTLQYPNALASFLVATSFTSFYLWGRLRTWPQFLLAAGNYVIVLVFLTTGSRGGYLVYPVTLILFFLLLPAGVRGKTLAHAAITFGGAFLANNFVIPAILAGNMGGAWAWFAAGLAVVEGAHVAWHLLARLTAGIPHRTRAIIGSGAVIAVLAVVAIFGAARFQAVPASTDQEGILARILPPTMLQRLQDINLETSGSAERLYWTQEAMKIVAAHPVLGAGGGAWEATYRSFQAYDYSSTQVHNHFAQIWSEIGTAGMAIWVGLWVLFLVTAAQAYRRSQGAARALPATLGVAGISLGMHAFIDFDLSLGAISLLLWSIWGLTRGLVAEKSQKSPGNRRLRLEWNWLWLVPFVAAGAIALFAASLLLGNYYARQAVAAATGGRAAEAGEYFRRASTFDPFTGSYLVDRAGLLVEKDPREAVRLARAAAEREPYNPRVLTRLGEIEWAAGHWPEAVAAMDKARDAAIWSVSGYENVGRVSALAAVNFLRQGDREQARQYAEKAAGIPAEMEERLAKVEPEARQLWQQSGRPFLAPSPGVMLNAGMAYFLLGKWDLARKSLETAAVDKGVGQEASLWLAALLEKTGDAAGAQKLLGEISRENQAWQQTYAFIHGLQVPL*

> NODE_66_length_50651_cov_165.733_41 UniRef100_A0A101ECR7 Fumarate reductase, flavoprotein subunit Taxonomy=Bacteria; Firmicutes; Clostridia; Thermoanaerobacterales; Thermoanaerobacteraceae; Moorella_group; Moorella

MQELSHDLLILGSGLAGLRAALEAARQTKGKLDIAIISKTQLMRSHSVCAEGGTAAALRPEEGDSLELHAWDTVKGSDFLADQDVVERFAAAMPGEILQLEHWGIPWSRKPDGRIDQRPFGGHSFDRAVYAADKTGFFEMQALYDTLQKYGGVVRYDEYYVTSILIENNRFCGLTFWNLATGEFGVIRGKALIIATGGACRIYGFTTYSYTVTGDGMAMAYRAGLPLKDMEFVQFHPTGLVPSGILITEAARGEGGYLTNNRGERFMKEYAASKIELAPRDIVARSEMIEIEQGRGFAGPRGLDYVNLDLRHLGADRINERLPLIREVAIKFNDIDPITAPIPVRPAAHYSMGGIHVNIDGKTPAEGIWAAGEAACISLHGANRLGANSTAECLVWGGITGGEVVRYLQHERHLPALPADRVREEEQRVFAGFFRRKGKENLYTIRQELRDLMDNKAGVFRTARELQEAQEEIRELGQRLQEAGLTDQSRIYNTDLISAIELENMLDLAQTIVAGALTREESRGGHARRDFPERDDSNWLKHTLAYYTPQGPRLEYIPVAITMWQPVERKY*

> NODE_66_length_50651_cov_165.733_42 UniRef100_A0A0T6ARF9 Uncharacterized protein Taxonomy=Bacteria; Armatimonadetes

MAEGRKRENYLGIKGWVRAGRYGLERYLYFLQRLTGFGLIIYLLIHLYETSFRLRGEVTWEGVMGLFDQPVFAVLEYVVMAAFIFHALNGLRLVIEELGFALGKPRRPVYPYETSLQRQRPFMWAIGVLIIVFLVVSLYDFLV*

> NODE_66_length_50651_cov_165.733_43 UniRef100_A0A0M2U3S6 Uncharacterized protein Taxonomy=Bacteria; Firmicutes; Clostridia; Clostridiales

MREAHFWLLQLVTGLVLIVLLGLHMIVIHLETILGFLGLGAGGAVAYASVMERAASSPWTWFYIIFLALALYHGLYGLRTIILELTLSRTASTVVTVLLVIVGIAAFSFGTYVTWQAYAG*

> NODE_66_length_50651_cov_165.733_44 UniRef100_A0A1F8M755 Succinate dehydrogenase iron-sulfur subunit Taxonomy=Bacteria; Chloroflexi

MFTEEQIITFKVQRFDPERDQGPYLKEYHVPYTQGLTVLDGLYYIKEKLDGSLAFRASCRMAVCGSCGMFINGKPHLACHTQITELESTVLEIRPLPNYSVIKDLVCDFTPLFAKHKAIKPYIIRREAGEIDNPTREFLQSPAELESYLQFSYCMKCGLCLAACPTAATDRNFLGPQALGQAYRYCADSRDDGLAERVKEIDHAHGVWRCHLAGACSEACPKGVDPALGIQLLKRQVVLRAVGLGKGRKLAAVVPPPSGQAKPRVEAPPFTVPRAKAEIAAGDSRS*

>NODE_66_length_50651_cov_165.733_45 # 47918 # 48745 # -1 # ID=6_45; partial=00; start_type=TTG; rbs_motif=AGxAGG/AGGxGG; rbs_spacer=5-10bp; gc_cont=0.606; ORF45; UniRef100_A0A1J4WUH9 Short-chain dehydrogenase n=1 Tax=Parcubacteria group bacterium CG1_02_41_26 TaxID=1805308 RepID=A0A1J4WUH9_9BACT

MKLDGKVAIITGAASGMGQAMALLFAREGATVVVADVNEVGGQETVRRIQEEARETSGQMERAAGAEARPTYGATDGRAEFVKVDVSQADQVKRLVDSTVNNYGKLNIMVNNAGVALLGKDGKIADVAEDTWDRVIAINLKGVYLGMKYAIPPMLKQGGGVIINTASIAGLVGFPSLAAYCASKGGIVQLTKATALDYGRDNLRVNAICPGVIRTAMTETMLADRETKEGMERTTPLPRLGEPEDIARAALYLASDEASFVTGTTLVVDGGWTAQ*

> NODE_66_length_50651_cov_165.733_45 UniRef100_A0A1J4WUH9 Short-chain dehydrogenase Taxonomy=Bacteria; unclassified_Parcubacteria_group

MFVRNHMSANPITVQPDDSINYAGTLMKEYGIRHLPVVSEGQLVGVVTQTDIFKVSASPATSLSIWELNYLIAKLAVKDAMTARVISVQEEAPVEEAALLMRQNKIGSLPVLNSTGKLVGIITETDLFDAILDAMGSNTATTRVVIECDDRPGELSRITGVIAEYGINIWSLVVFHPAEGIAHVVVRLQGENLEGVFEKLASEDLRLVR*

>NODE_66_length_50651_cov_165.733_46 UniRef100_UPI0004712E7E transcriptional regulator Taxonomy=Bacteria; Firmicutes; Clostridia; Clostridiales; Caldicoprobacteraceae; Caldicoprobacter

MLSEVREEMVAVIADVLSDGRARTLEQVLAELRAEYPESVETASCEYASAYGYSGCGQLMAPVNAVADALACLEGRGEAVSFFRDGLKLWQNAS*
